# Topological linking determines elasticity in limited valence networks

**DOI:** 10.1038/s41563-024-02091-9

**Published:** 2025-01-31

**Authors:** Giorgia Palombo, Simon Weir, Davide Michieletto, Yair Augusto Gutiérrez Fosado

**Affiliations:** 1 School of Physics and Astronomy, University of Edinburgh, Edinburgh, UK; 2 MRC Human Genetics Unit, Institute of Genetics and Cancer, University of Edinburgh, Edinburgh, UK

**Keywords:** Topological matter, Gels and hydrogels, DNA and RNA

## Abstract

Understanding the relationship between the microscopic structure and topology of a material and its macroscopic properties is a fundamental challenge across a wide range of systems. Here we investigate the viscoelasticity of DNA nanostar hydrogels—a model system for physical networks with limited valence—by coupling rheology measurements, confocal imaging and molecular dynamics simulations. We discover that these networks display a large degree of interpenetration and that loops within the network are topologically linked, forming a percolating network-within-network structure. Below the overlapping concentration, the fraction of branching points and the pore size determine the high-frequency elasticity of these physical gels. At higher concentrations, we discover that this elastic response is dictated by the abundance of topological links between looped motifs in the gel. Our findings highlight the emergence of ‘topological elasticity’ as a previously overlooked mechanism in generic network-forming liquids and gels and inform the design of topologically controllable material behaviours.

## Abstract (web summary)

Experiments and simulations of DNA nanostar hydrogels reveal that microscopic topology determines macroscale elasticity in amorphous networks.

## Main

Networks made by colloids and polymers are fundamental components of virtually any material around us^1^, and even a substance as familiar as water exhibits complex network structures^2,3^. Understanding the relationship between the microscopic structure of these networks and their mesoscopic and macroscopic material properties has been the focus of the material science community for decades. Classic textbook pictures mainly focus on entanglements^4^ and crosslinks^1^ to predict the material properties of gels and complex fluids; however, the role played by network motifs that impose formal topological invariants is far less understood^5^.

Most classic theories rely on chemical composition, fraction of branching points, mesh size and fractal dimension to predict the mechanical properties of soft colloidal and polymeric gels^6^. However, it is becoming apparent that to accurately describe a material’s behaviour, we also require a quantitative understanding of rigorously defined topological motifs created by the microscopic building blocks, such as loops, knots and links. This need is especially evident in, for example, gels with looped defects^7–9^, solutions of ring polymers^10–13^, Olympic gels^14–16^, molecular knots^17^, polyrotaxanes^18^, soft particulate gels^19^ and even associating liquids, such as water^2,3^.

Especially challenging are physical networks with limited valence, where the connectivity of the building blocks is constrained and the networks are thus expected to display unconventional structures^2,20–22^. Experimentally, limited valence networks are most commonly found in associating liquids^2^, but are best investigated at larger timescales and length scales through model systems made of patchy colloids^23,24^ or DNA nanostars^25^ (DNAnss), where single-stranded DNA oligomers are self-assembled into star-shaped building blocks with a fixed number of arms and terminal ‘sticky ends’ forming reversible (or physical) networks^25–27^. DNAnss self-assemble into complex fluids and physical gels with a range of viscoelastic properties, which have been studied extensively in recent years^21,25,27^.

**Fig. 1:**
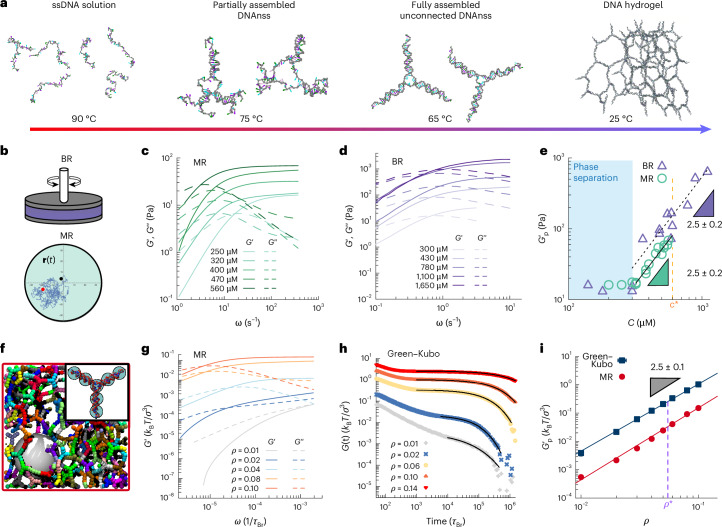
Scaling of elasticity in gels of DNAnss. **a**, Schematic of the annealing procedure for assembling individual DNAnss and the full hydrogel. ssDNA, single-stranded DNA. **b**–**e**, BR and MR results at different DNAns concentrations. **c**,**d**, Elastic (*G*′(*ω*)) and viscous (*G*″(*ω*)) moduli from MR (**c**) and BR (**d**). **e**, Elastic plateau ${G}_{{\mathrm{p}}}^{{\prime} }$ as function of DNAns concentration (also Supplementary Fig. 2 with errors in the concentration of DNAnss). The cyan-shaded area represents the concentrations at which the system phase separates^52^. Data is well fitted by a power law ${G}_{{\mathrm{p}}}^{{\prime} } \propto {C}^{2.5}$ in the gel region for both techniques (black lines). Labels give best-fit exponents with standard fitting error estimates. Orange dashed line indicates the overlapping concentration c* = 600 μM (Supplementary Section VI). Triangles are used to illustrate scalings. **f**, Schematic of the simulated coarse-grained DNAns hydrogel and its assembly into a network. A large bead (white) is used to simulate MR. **g**, *G*′ and *G*″ from MR simulations. **h**, Stress-relaxation function from equilibrium Green–Kubo simulations. Black lines are fits of a stretched exponential function to the data (Supplementary Fig. 6 for details). **i**, Elastic plateau (${G}_{{\mathrm{p}}}^{{\prime}}$) as a function of the volume fraction *ρ* from simulated MR and the Green–Kubo relation (Supplementary Section V for details). Purple dashed line indicates the overlapping volume fraction ρ* = 0.056. Data points represent mean ± s.e.m. (smaller than symbol size) from five independent replicates. Data are well fitted by a power law ${G}_{{\mathrm{p}}}^{{\prime}} \propto {\rho }^{2.5}$ in the whole range of concentrations. Triangles are used to illustrate scalings.

We consider DNAns gels as model systems to study generic networks formed by limited valence building blocks (Fig. 1a). Although the operational definition of a gel is that its elastic response dominates at arbitrarily low frequencies, here we adopt the terminology used in the literature^21,25,27^. We thus refer to ‘DNAns gels’ and to their elasticity, specifically in the context of the high-frequency regime, where their behaviour is elastic-dominated. DNAns gels are in fact complex fluids that display a Maxwellian behaviour, that is, elastic at large frequencies but liquid at long enough timescales^21,25,27,28^. Gaining a better understanding of DNAns hydrogel rheology will speed up the bottom-up discovery and formulation of new DNA-based soft materials.

We couple extensive molecular dynamics (MD) simulations of an oxDNA-inferred^29^ coarse-grained model of DNAnss, with bulk and microrheology (MR) experiments and confocal imaging, to elucidate the connection between network structure and, more importantly, topology (such as knots and links), and the network’s elastic behaviour. By spanning a range of concentrations *C*, we discover that the system undergoes a topological transition around the overlapping concentration c*, where loops within the network start to link with each other. While for *C* < c* the fraction of branching points and the mesh size of the network (that is, its structure) govern the elasticity of the gel, for *C* ≥ c*, linking of loops within the network (that is, its topology) emerges as the main determinant of the gel’s mechanical properties. In the latter regime, the elasticity of the gel, that is, its solid-like response to high-frequency stress, scales with the concentration of DNAnss as ${G}_{{\mathrm{p}}}^{{\prime} } \propto {C}^{2.5}$ and scales linearly with the linking number between minimum loops, ${G}_{{\mathrm{p}}}^{{\prime} } \propto {\mathcal{L}}$, where ${G}_{{\mathrm{p}}}^{{\prime} }$ is the elastic plateau, i.e., the value of the elastic modulus at the largest frequency.

This simple relationship connects the macroscopic mechanical properties of a soft material with a mathematically rigorous topological invariant that is markedly distinct from generic ‘entanglements’ or crosslinks.

## Scaling of elasticity in physical gels with limited valence

We first assembled tri-armed DNAnss as done previously (Supplementary Section I and the literature^21,25^). We then prepared solutions at different concentrations by annealing the DNAnss via quenching from 90 °C to 25 °C (Fig. 1a and Supplementary Section II). Particle tracking MR and oscillatory bulk rheology (BR) were then employed to measure the frequency-dependent elastic modulus (*G*′(*ω*)) and viscous modulus (*G*″(*ω*); Fig. 1c,d and Supplementary Section III). In Supplementary Fig. 2, we also show that *G*′ and *G*″ from different concentrations superimpose into a master curve that follows a near-Maxwellian behaviour with a characteristic unbinding time ${\tau_{u}\propto} {\omega }_{0}^{-1}$ (with *ω*_0_ the cross-over frequency at which *G*′ = *G*″), due to the reversible nature of the hybridization between DNAnss.

We extracted the elastic plateau, ${G}_{{\mathrm{p}}}^{{\prime} }$, as the value of the elastic modulus at the largest frequency *ω* ∝ 10^2^ Hz (although we obtained similar results by choosing the value of *G*′ at the crossover point; Supplementary Section III). The measurements from BR and MR are in very good agreement, yielding a scaling ${G}_{{\mathrm{p}}}^{{\prime}} \propto {C}^{2.5\pm 0.2}$ at *C* > 300 μM (beyond the phase-separation region; Fig. 1e), in agreement with values obtained for other limited valence gels^30^. The values of ${G}_{{\mathrm{p}}}^{{\prime} }$ in BR are consistently larger than the ones found in MR, likely due to surface effects between the DNA and the probe particle^31^ (Supplementary Fig. 4). While the scaling exponent found here is larger than the one reported in the literature for a similar system, both the range of elasticity (${G}_{{\mathrm{p}}}^{{\prime} } \approx 10{-}700 \ {\mathrm{Pa}}$) and relaxation timescales (*τ*_u_ ≈ 1 s) are in line with those in the literature^21^. To better understand how this scaling emerges from the network structure, we turned to MD simulations.

## Coarse-grained model captures bulk behaviour of DNA networks

We used a recently developed computational coarse-grained model of rigid analogues of DNAnss^29^ with valence *f* = 3 (Fig. 1f). Briefly, each nanostar is modelled as a rigid body with a Y-shaped structure that was inferred from oxDNA simulations^29,32^ (Methods and Supplementary Section IV). We simulate solutions of DNAnss at different volume fractions, ranging from *ρ* = 0.01 to 0.14. To measure the viscoelasticity, we first track the position (**r**) of a large bead embedded in the sample and compute its mean squared displacement at a lag-time *t*, MSD(*t*) = 〈∣**r**(*t* + *t*_0_) − **r**(*t*_0_)∣^2^〉, where the average is performed over different values of the starting time *t*_0_. We use the generalized Stokes–Einstein relation to compute the complex stress modulus^33^ and the elastic plateau ${G}_{{\mathrm{p}}}^{{\prime} }$ (Fig. 1g and Supplementary Section V). In parallel, we also use the Green–Kubo relation and compute the autocorrelation of the off-diagonal components of the stress tensor (*P*_αβ_ = *P*_xy_, *P*_xz_ and *P*_yz_; with *x*,*y* and *z* the spatial directions) to obtain the stress-relaxation function^34,35^ $G(t)=\frac{{L}^{3}}{3{k}_{{\mathrm{B}}}T}{\sum }_{\alpha \ne \beta }{P}_{\alpha \beta }(0){P}_{\alpha \beta }(t)$ (where *L* is the size of the box, *k*_B_ is the Boltzmann constant and *T* the temperature of the system; Methods for details). The elastic plateau of the system is then obtained by fitting a stretched exponential function $g(t)=a\exp (-{(t/\tau )}^{b})$, in which *a*, *τ* and *b* represent the elastic plateau, the relaxation time of the network and the stretched exponent, respectively^36^ (Fig. 1h). Both methods are in excellent agreement and yield a network elasticity that scales as ${G}_{{\mathrm{p}}}^{{\prime} } \propto {\rho }^{2.5\pm 0.1}$ (Fig. 1i), exactly matching the one found in our experiments. In fact, we even obtain the same result with a more flexible DNAns model (Supplementary Fig. 18). Thus, we argue that our simulations capture the behaviour and internal microstructure of the gels well. In line with the literature^21^, our simulations suggest that the gels’ elasticity displays a nonlinear scaling with concentration, different from predictions of classic models, such as phantom network theory. To understand the underlying physical origin of elasticity in these networks, we thus now investigate the microscopic properties and topology of our simulated DNAns gels.

## DNAns gel length scales suggest network interpenetration

**Fig. 2:**
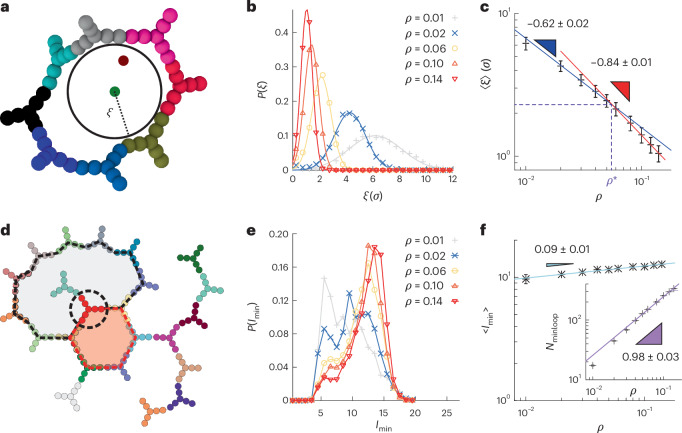
Characterization of geometrical features in simulated DNAns networks. **a**, Schematic of the random insertion method^37^: the largest sphere with radius *ξ* containing a random point (in red) is shown. **b**, Probability distribution of the mesh size *ξ*. Lines are fits to the data using a Gaussian function. The mes size is given in simulation units, where *σ* = 2.5 nm represents the diameter of a bead (Supplementary Section IV). **c**, Mean and s.e.m. of the mesh size obtained from the previous fit, using 50 independent gel configurations per volume fraction. Lines represent a power law fit to the data from which labels with the best-fit exponents are obtained. Triangles are used to illustrate scalings. **d**, Sketch of the minimum loop search algorithm. We highlight the two loops passing through the red DNA nanostar (shaded grey and red). The minimum loop, with *l*_min_ = 6 nanostars, is shaded red. **e**,**f**, Probability distribution of the number of nanostars in the minimum loops *l*_min_ (**e**). Lines are for visual guidance. The mean and s.e.m. of the distribution at different concentrations are used to show the *l*_min_ scaling with *ρ* in **f**. Inset shows scaling of the number of minimum loops with volume fraction. Error bars are smaller than symbols. Results in this panel use at least 25 independent gel configurations per volume fraction. Supplementary Section VII and Supplementary Figs. 9 and 10 have more details and the scaling of distance between branch points. Lines represent a power law fit to the data from which labels with the best-fit exponents are obtained. Triangles are used to illustrate scalings.

One of the most important length scales in polymers and colloidal solutions is the mesh size (*ξ*), or the size of the gel pores. We computed the mesh size in our simulated gels by a method of random insertions^37^, done by randomly choosing a point in our simulation box and by finding the largest sphere that satisfies two conditions: containing the point and not touching any of the nanostar beads (Fig. 2a). We repeat this procedure 200,000 times over 50 independent gel configurations and obtain the probability distribution (*P*(*ξ*)) of the mesh size, that is the diameter of the space-filling sphere, at different volume fractions (Fig. 2b). We find Gaussian-distributed values, from which we extract the average mesh size 〈*ξ*〉 (Fig. 2c). Interestingly, at the overlap concentration, ρ* = 0.056, we identify a transition where the scaling of 〈*ξ*〉 changes from *ξ* ∝ *ρ*^−0.62^ to *ξ* ∝ *ρ*^−0.84^ (Supplementary Section VI). We interpret this as a sign of a structural transition, suggesting the onset of a network geometry that allows for tighter packing. This length scale appears to have a similar scaling to that measured in strain hardening experiments^21^.

Another key structural feature in gels is the presence of loops within the network. While loops are often associated with weak motifs (or defects) in polymer networks as they reduce the density of ‘active’ crosslinks^38^, we argue that in the case of branched, amorphous networks, short loops may also act as topological springs. To quantify the abundance of loops within the network, we employed graph analysis (Supplementary Section VII), focusing on so-called ‘minimum’ loops, which are computed as follows: for each DNAns (for example, the one circled with dashed lines in Fig. 2d) we found all the loops passing through it and selected the loop with the least number (*l*_min_) of DNAnss (highlighted by the red shaded area in Fig. 2d). We then compiled the statistics of all minimum loops in the network and calculated the probability *P*(*l*_min_) that a minimum loop is formed by *l*_min_ nanostars and the first momentum 〈*l*_min_〉 of the distribution (Fig. 2e,f), which we found has values similar to the estimated cluster size in the literature^21^. Remarkably, these distributions shift to larger values of 〈*l*_min_〉 for larger concentrations of DNAnss, indicating that the average minimum loop length increases with DNAns concentration. This is in marked contrast with the finding above, that is, that the mesh size decreases with DNAns concentration. Indeed, although the scaling of *l*_min_ is weak, with 〈*l*_min_〉 ∝ *ρ*^0.1^, the number of minimum loops (*N*_minloop_) changes with *ρ* (Fig. 2f, inset).

Additional quantities that typically characterize the mechanical properties of gels are (1) the number of branching points and (2) the distribution of the shortest path (*λ*) connecting two branching points, *P*(*λ*) (ref. ^39^). In marked contrast with typical results from chemical crosslinking gels, we discover that the average path length 〈*λ*〉 increases with DNAns concentration, with a similar exponent found for the growth of minimum loop length (Supplementary Section VII). Importantly, we also find that the fractal dimension of the gel (*d*_f_ = 1/*ν* = 1.7, with *ν* = 0.58, the exponent relating the radius of gyration and the number of DNAnss of the shortest paths) is independent of the concentration of DNAnss, suggesting that the transition from liquid to gel behaviour must be accompanied by a distinct type of transition in the network that does not affect the geometric and fractal arrangement of the nanostars (Supplementary Fig. 9).

**Fig. 3:**
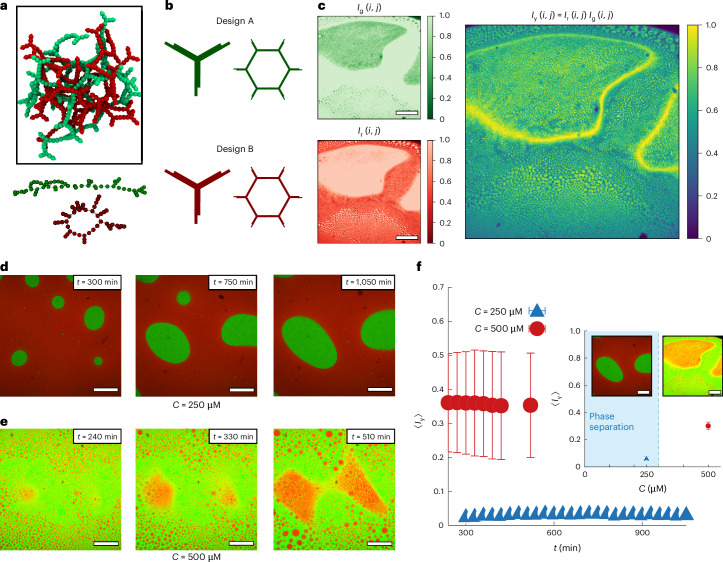
Interpenetration of DNAns networks. **a**, Snapshot from simulations performed with only one type of DNAns at *ρ* = 0.04 displaying interpenetrated structures in a three-dimensional spherical neighbourhood of the simulation box, with the corresponding disjoint graph diagrams at the bottom (we found similar results in simulations of a binary mixture of DNAnss; Supplementary Fig. 17). **b**, Schematic representation of DNAns designs for the experimental interpenetration assay. The cores of DNS-A (green) and DNS-B (red) have the same nucleotide composition but variations in their sequence that avoid accidental cross-hybridization. The two designs have orthogonal overhangs with the same lengths and interaction strengths, allowing for A–A and B–B interactions but no A–B hybridization. **c**, Confocal images showing partial interpenetration when mixing [DNS-A] = 500 μM and [DNS-B] = 500 μM. In the left panel, we show images in the two channels: FAMK (488 nm, green, top) and Cy3 (555 nm, red, bottom). The right panel is the pixel-by-pixel product of the images, or the yellow signal, *I*_y_(*i*, *j*) = *I*_r_(*i*, *j*)*I*_g_(*i*, *j*). **d**,**e**, Confocal images at different times for systems at 250 μM and 500 μM, respectively. Note that the ones at 500 μM display growing red patches that are themselves filled with green droplets even after several hours. The two sample mixtures were prepared from the same pure batches of DNS-A and of DNS-B, following the protocol in Supplementary Section VIII. **f**, Temporal evolution of the intensity of the yellow signal. Each data point represents the mean ± s.d. of the yellow intensity across the image (calculated at a fixed time point). The inset shows the mean ± s.d. of yellow intensity for each concentration averaged over all the displayed time points. We also show representative confocal images from experiments in the respective phases, with scale bars of 250 μm for all images.

To summarize, our analysis from simulations suggests that as one increases the concentration of building blocks in limited valence gels, the microstructure displays the formation of longer (and larger) loops but tighter pore sizes. We realized that these two seemingly contrasting behaviours can be reconciled if these networks formed interpenetrating structures. Indeed, interpenetration would reduce the space in between DNA strands, yet require longer loops to accommodate loop threading. Motivated by this conjecture, we set out to detect the presence of interpenetration in our simulations: we selected all nanostars within different spherical neighbourhoods in our simulations and created adjacency graphs. We discovered that above ρ* most subsets form two unconnected graphs and show the presence of interpenetrated structures (Fig. 3a). Our evidence strongly suggests that generic limited valence networks form interpenetrating networks, leading us to anticipate the presence of topologically complex motifs. While this picture is similar to the one discovered in the hydrogen bond networks of water^2^ and ice^3^, we argue that in DNAns networks, these topological motifs may play a role in determining the gel’s material properties.

## Confocal imaging supports interpenetration in DNAns gels

To experimentally test interpenetration in DNAns hydrogels, we designed two types of nanostars (DNS-A and DNS-B) that have the same geometry and nucleotide composition but enough variations in their core sequences to avoid misfolding and unintended A–B crosslinking^40^ (Fig. 3b and Supplementary Table 3). The overhang sequences employed for the two designs are self-complementary but distinct for the A and B nanostars, so that only A–A or B–B hybridization is enthalpically favoured and with equal self-binding strength (Supplementary Section VIII). To identify each design, we introduced modified nucleotides bound to fluorophores—FAMK for DNS-A and Cy3 for DNS-B—in the middle of one of the double-stranded DNA arms of the nanostars.

Upon mixing equimolar amounts of DNS-A and DNS-B, each beyond their own gel binodal, microemulsions are formed: small A-rich droplets form in a large B-rich droplet, and vice versa. We multiply the normalized green *I*_g_ and red *I*_r_ signal intensities in each pixel to obtain a two-dimensional map of ‘yellow’ pixels with intensity *I*_y_(*i*, *j*) = *I*_r_(*i*, *j*) × *I*_g_(*i*, *j*) (Fig. 3c). A large value of *I*_y_ indicates the presence of both DNS-A and DNS-B, which we identify as an interpenetrated region.

We tracked the temporal evolution of confocal images from experiments at two different nanostar concentrations. At *C* = 250 μM, within the phase-separation region, samples are observed to completely demix over time (Fig. 3d,f). Beyond the phase-separation region (at *C* = 500 μM), we observed that mixed ‘yellow’ regions are stable over hours and even weeks (Fig. 3e,f and Supplementary Fig. 14). To quantify the extent of interpenetration as a function of DNAns concentration, we computed the average intensity of *I*_y_(*i*, *j*) over time using more than 20 representative images taken at long times, to obtain 〈*I*_y_〉. In the phase-separation region, we observed fully demixed phases and 〈*I*_y_〉 ∝ 0 (Fig. 3f). Using the same microscope settings, gel samples show a considerably larger value, on average 〈*I*_y_〉 ∝ 0.4. These observations suggest the presence of partially interpenetrated structures in DNAns hydrogels, in line with indirect evidence from dynamic light scattering obtained in a different DNAns hydrogel design^41^.

We note that while yellow regions (demarcating a high local density of both DNS-A and DNS-B) confirm the interpenetration between the two networks, darker regions (with an unbalanced density of DNS-A and DNS-B) may still form interpenetrated structures made by mainly one type of DNAns. Therefore, we have verified (Supplementary Fig. 15) that the intensity of the individual signals, either red or green, is lower (higher) in the balanced (unbalanced) regions. We have also confirmed through MD simulations that a binary mixture of DNAnss yields the same interpenetrated structures and scaling of elasticity as the monodisperse system (Supplementary Fig. 17).

## Characterizing topology motifs in interpenetrated networks

**Fig. 4:**
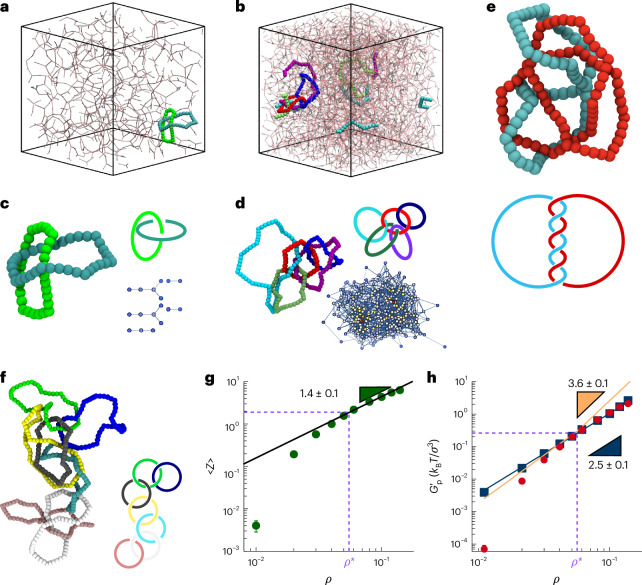
Topological elasticity. **a**,**b**, Representative snapshots from simulations of networks at *ρ* = 0.02 (with *N*_minloop_ = 47 and ${\mathcal{L}}=12$) and *ρ* = 0.1 (with *N*_minloop_ = 256 and ${\mathcal{L}}=1{,}159$), respectively. **c**,**d**, Example of linked rings found in the snapshots from **a** and **b**. Network diagrams represent minimum loops as nodes and linkages between loops as edges (unlinked minimum loops not shown). **e**,**f**, At high concentration (*ρ* = 0.1), more complex topologies are observed: minimum loops with linking number 3 (**e**) and polycatenanes (**f**). **g**, Average number of links per minimum loop, that is, linking valence, as a function of concentration. Error bars are smaller than symbols. Triangle are used to illustrate scalings. **h**, Scaling of elasticity with concentration comparing Green–Kubo simulations (dark blue, with *G*′ ≈ *ρ*^2.5^ in the whole range of concentrations; also Fig. 1i) and predictions from (1) mesh size (orange line) and (2) linking number (red circles). The last two quantities have been multiplied by 0.03 and 0.001, respectively, to help the comparison of the scaling. In **g** and **h**, values are expressed as mean ± s.e.m., obtained from at least 25 independent configurations per volume fraction. Triangles are used to illustrate scalings.

Having found strong evidence for the emergence of interpenetrated structures in both simulations and experiments, we decided to characterize the ensuing topological motifs. Typical configurations of the networks formed at two volume fractions, *ρ* = 0.02 and *ρ* = 0.1, are shown in Fig. 4a,b, where it is clear that as the concentration of DNAnss increases, topological links between the minimum loops appear to be more abundant in the network.

We then computed the Gauss linking number (Lk) between all possible pairs of minimum loops in the network as follows:

1 $$\,\text{Lk}\,({\gamma }_{i},{\gamma }_{j}^{{\prime} })=\frac{1}{4\pi}{\oint }_{{\gamma }_{i}}{\oint }_{{\gamma }_{j}^{{\prime} }}{\mathrm{d}}s\,{\mathrm{d}}{s}^{{\prime} }\frac{({\bf{t}}(s)\times {\bf{t}}({s}^{{\prime} }))\cdot ({\bf{r}}(s)-{\bf{r}}({s}^{{\prime} }))}{| {\bf{r}}(s)-{\bf{r}}({s}^{{\prime} }){| }^{3}},$$

where *γ*_*i*_ and ${\gamma }_{j}^{{\prime} }$ are loops formed by fragments of DNAnss, **t**(*s*) is the tangent at position *s* along the loop and **r**(*s*) is the three-dimensional coordinate of the segment at position *s* along the loop. Strikingly, we discovered that the fraction of minimum loops that are linked (at least once) grows from 0 at *ρ* = 0.01 to 95% at the overlap concentration ρ* = 0.056. Additionally, at large concentrations, we observed the emergence of complex topologies, such as multiply linked loops (Fig. 4e) and polycatenanes (Fig. 4f). Fascinatingly, these interlinked loops themselves form a percolating network (Fig. 4d, inset), thus establishing a network-within-network structure in DNAns gels.

Since DNAnss interact through physical (reversible) bonds, the minimum loops are not permanent and we observed that the system’s topology changes over time. In our simulations, the networks reach a steady state in which the total number of minimum loops and total linking (${\mathcal{L}}=\mathop{\sum }\nolimits_{i\ > \ j}^{{N}_{{\mathrm{minloop}}}}| \,\text{Lk}\,(i,j)|$) fluctuate around constant values (Supplementary Figs. 10e and 11c). The average linking number per (minimum) loop, that is, the average linking valence $\langle \,{Z}\,\rangle ={\mathcal{L}}/{N}_{{\mathrm{minloop}}}$, as a function of the concentration of DNAnss, is shown in Fig. 4g. Interestingly, the overlap concentration ρ* marks the point at which the system undergoes a change in its topological state: at *ρ* < ρ*, the linking between loops is negligible (〈*Z*〉 < 1), while for *ρ* ≥ ρ*, all loops are on average linked at least once (〈*Z*〉 > 1). We also note that the average linking valence grows as 〈*Z*〉 ∝ *ρ*^1.4^, which is consistent with a simple geometrical consideration on the scaling of the number of overlapping minimum loops, expected to follow $\langle Z\,\rangle \approx {N}_{{\mathrm{minloop}}}{R}_{{\mathrm{g,min}}}^{3}/V \approx \rho {\langle {l}_{{\mathrm{min}}}\rangle }^{3} \propto {\rho }^{1.3}$ (where *R*_g,min_ is the average radius of gyration of minimum loops, *V* is the total volume of the simulation box and we used 〈*l*_min_〉 ∝ *ρ*^0.1^; Supplementary Section VII and Fig. 2f).

This topological transition in the linking of (minimum) loops at ρ* explains the counterintuitive results seen above: as the concentration of DNAnss increases, not only do more loops appear in the network, but these loops become larger concomitantly with the emergence of more tightly packed, interpenetrated structures, which in turn favour the formation of links and increase the topological complexity of the network.

We argue that at timescales shorter than the typical unbinding time of individual DNAnss (in our DNAns design, *τ*_u_ ∝ 1 s at 25 °C), perturbations to the network will effectively apply stress to these topological interlocked features as if the network topology was frozen. Therefore, the topology of interlinked minimum loops would determine the elastic response of the gel at frequencies $\omega > {\tau }_{{\mathrm{u}}}^{-1}$. Given that our elastic behaviour was measured around *ω* ∝ 100 Hz, which is ${\sim} 10\,{\tau }_{{\mathrm{u}}}^{-1}$ (Fig. 1), we expect the scaling of ${G}_{{\mathrm{p}}}^{{\prime} }$ to be determined by the linking of the minimum loops.

## Topological elasticity

The elasticity of polymer networks can be understood as the combined effect of the density of crosslinks (related to the network connectivity) and that of entanglements^42,43^. At high crosslinking density, crosslinks dominate over entanglements, and classical theories, such as the affine and phantom network models, neglect excluded volume interactions between polymers and the presence of entanglements^44^. Indeed, for building blocks with limited valence *f*, phantom network theory predicts ${G}_{{\mathrm{p}}}^{{\prime} } \propto \rho (\;f-2)/f$. Simulations of disordered phantom networks showed that this simple relation fails to predict the elasticity of the system, which is instead better described by an even weaker scaling, ${G}_{{\mathrm{p}}}^{{\prime} } \propto {\rho }^{1/3}$ (ref. ^45^). Thus, current models fail to predict the nonlinear scaling of the elastic plateau with concentration that is observed in our simulations and experiments, ${G}_{{\mathrm{p}}}^{{\prime} } \propto {\rho }^{2.5}$ (Fig. 1e,i). Large scaling exponents for the elastic behaviour at large frequencies were also found in previous works on similar complex fluids made of limited valence building blocks^21,46^. A comprehensive and predictive theory for the behaviour of limited valence reversible gels should account for the topological features we uncovered and reported in the previous sections. Specifically, we note that for *ρ* ≥ ρ*, if the elasticity at high frequency was determined by the mesh size, we would expect it to phenomenologically scale as follows (Supplementary Section VI):

2 $${G}_{{\mathrm{p}}}^{{\prime} } \propto \frac{{k}_{{\mathrm{B}}}T{N}_{{\mathrm{minloop}}}}{{\xi }^{3}} \propto {\rho }^{3.6}\,,$$

where we made use of the fits *N*_minloop_ ∝ *ρ* and *ξ* ∝ *ρ*^−0.84^ at large *ρ* (Fig. 2). This scaling is shown in Fig. 4h to markedley deviate from direct Green–Kubo measurements at large *ρ* (Fig. 4h, blue squares).

Instead, we find that the scaling of the Green–Kubo-measured elasticity is better explained by the following simple argument: assuming that linking between minimum loops becomes the dominant entanglement mechanism on timescales shorter than the typical melting time of the sticky ends, the high-frequency elastic plateau should be proportional to (1) the density of elastically active polymers *ρ*_e_ (the density of minimum loops) and (2) the number of entanglements per polymer *Z*_e_ (the average linking valence). We thus expect

3 $${G}_{{\mathrm{p}}}^{{\prime} } \propto {\rho }_{{\mathrm{e}}}{\text{Z}}_{{\mathrm{e}}} \propto {N}_{{\mathrm{minloop}}}\langle \,{Z}\,\rangle \propto {\mathcal{L}} \propto {\rho }^{2.4}\,,$$

where we made use of *N*_minloop_ ∝ *ρ* and 〈*Z*〉 ∝ *ρ*^1.4^ (Fig. 4g). This scaling is in very good agreement with both experiments and simulations (Fig. 4h). The small discrepancy in the predicted exponent (2.4) and the one measured through Green–Kubo calculations (2.5) is within uncertainty. This discrepancy could also be attributed to the fact that our topological analysis neglects links between second-order loops (larger than the minimum ones) and it also ignores loop threading.

## Conclusions

In this paper we have tackled a long-standing question: the connection between a material’s microscopic topology and its macroscopic material properties. We focused on soft viscoelastic gels made of limited valence building blocks with transient, non-covalent crosslinks and realized them in the lab using tri-armed DNAnss.

The central conclusion drawn from our findings is that at timescales shorter than the DNAns sticky-end unbinding, the elastic response of the gel is dominated by linked loops, akin to Olympic networks^1,14^ like kinetoplast DNA^16,47,48^. In fact, the linking of minimum loops grows as ${\mathcal{L}} \propto {N}_{{\mathrm{minloop}}}\langle Z\,\rangle \propto {\rho }^{2.4}$, matching the scaling of the elastic plateau (Fig. 4). The simple linear relationship found (${G}_{{\mathrm{p}}}^{{\prime} } \propto {\mathcal{L}}$) is very powerful as it connects the rheology of the network with its topology (in the formal mathematical sense of the term, not referring to generic entanglements).

Based on our findings, we argue that if we were to design DNAnss with longer arms, or to use binary mixtures of DNAnss with each type having arms of uniform length and with the arm lengths differing between the two types, we would obtain stronger networks, despite the effective reduction of crosslinks per unit volume. Additionally, by designing DNAnss with longer sticky ends (and hence longer unbinding times) we would realize semi-irreversible interactions, and complex fluids with an elastic behaviour at lower frequencies. In fact, through DNA ligation we could render DNAns–DNAns bonds irreversible. Whether the ensuing low-frequency elastic behaviour could be described by the linkages between minimum loops in the network is to be determined, as the gelation kinetics may also contribute to affect the scaling.

We expect that our results should hold for other network-forming structures made by limited valence building blocks, as they favour the formation of complex porous networks. We expect that the ‘topological elasticity’ mechanism uncovered in this paper will be relevant to understand the physical properties of generic networks covering different time and length scales and made by, for instance, patchy colloids^46,49^, polymers^38^, molecular liquids such as water and ice^2,3^, DNA^21,25,27^, polymer rings^10,13,14,50^ and potentially other complex fluids such as polymer gels and liquid crystals with entangled defects^30,51^. Indeed, topology is by its nature universally found across physical and biological systems. Overall, our findings contribute to a better fundamental understanding of how formal topological motifs within a network affect the material properties of the bulk, and will also guide the design of topologically controlled structural and mechanical properties of generic network-forming liquids and materials.

## Methods

### DNAns design

Nanostar motifs were assembled from three single-stranded oligonucleotides. Each oligo was 49 nucleotides long and had sequences as reported in Supplementary Table 1. These motifs were designed using NUPACK^53^, with minor modifications from those originally proposed in ref. ^26^. Each double-stranded DNA arm was 20 base pairs long, and terminated in a self-complementary six-nucleotide fragment with sequence 5′-CGATCG-3′. This sticky end was equal for all three arms, allowing the non-specific hybridization of two nanostars: any of the three arms of one nanostar could hybridize with any (but only one) of the arms of another nanostar. Unpaired adenines (A) were introduced at the core of the Y-shaped structure and before the sticky end to enhance the internal flexibility of the nanostar and the nanostar–nanostar bond. The nucleotide sequences for the three-armed nanostar were acquired from Integrated DNA Technologies (IDT, https://www.idtdna.com/pages). Oligonucleotide tubes were then dissolved in UltraPure DNase/RNase-Free Distilled Water (Invitrogen, 11538646) and used to form nanostar samples as described in Supplementary Section II. Nanostar samples were prepared at the desired concentration in Nanostar Buffer, containing the following reagents: Tris (Invitrogen, 15504020), sodium acetate (Sigma-Aldrich, 33209-M), ethylenediaminetetraacetic acid (Sigma-Aldrich, ED2P) and NaCl (Thermo Scientific Chemicals, 10092740).

### Microrheology

We used particle tracking MR to assess the mechanical properties of samples of nanostars at different concentrations. Polystyrene beads of 200 nm size (Sigma-Aldrich, 69057) were spiked in the solution and tracked by using trackpy (github.com/soft-matter/trackpy). The viscoelastic properties of the sample were then obtained by applying the generalized Stokes–Einstein relation^54^ to the mean squared displacement of the beads (Supplementary Section III and Supplementary Fig. 1 for details).

### Bulk rheology

Frequency sweeps were performed at a shear strain of *γ* = 0.5% within the linear viscoelastic region. DNAns solutions behave as near-Maxwellian viscoelastic fluids, with low-frequency liquid behaviour (*G*″ > *G*′, with *G*′ ∝ *ω*^2^ and *G*″ ∝ *ω*) separated by a crossover frequency (*ω*_0_) from high-frequency solid-like behaviour (*G*′ > *G*″) and with a plateau modulus ${G}_{{\mathrm{p}}}^{{\prime} }$ (Supplementary Section III and Supplementary Fig. 2 for details).

### Confocal imaging

DNS-A and DNS-B solutions were prepared in two separate test tubes to prevent the formation of unintended secondary structures during the annealing step. In both cases, the fluorescently tagged and untagged oligomers (purchased from IDT, with sequences reported in Supplementary Table 3) were mixed at a molar ratio of 1:10 (or 1:20). Samples were visualized with a Zeiss LSM700 confocal microscope with a scan time of 15.49 s and a laser intensity equal to 2.4% (no bleaching effects were detected at this intensity value). During the image acquisition, the 488 nm and 555 nm lasers scan the sample sequentially to excite FAMK (DNS-A, green) and Cy3 (DNS-B, red). To visualize the final image, we assembled the two channels into one using Fiji (Supplementary Section VIII and Supplementary Fig. 14 for details).

### Coarse-grained model and simulations

In simulations, seven beads were assembled into a Y-shaped rigid structure that resembled a nanostar. Small patches were placed at the end of each arm to represent sticky sites. The core particles were purely repulsive, with an excluded volume of *σ* = 2.5 nm ∝ 8 base pairs. The patches interacted via a short-range attractive Morse potential (Supplementary Information equation (2) and Supplementary Section IV), and the geometry of the bead-patch and parameters of the Morse potential were set to ensure one-to-one binding of the simulated nanostars, thereby mimicking sticky-end hybridization. MD simulations were performed using the large-scale atomic/molecular massively parallel simulator (LAMMPS) and in the *NVT* ensemble, for which the number of particles (*N*), volume (*V*) and temperature (*T*) were held constant. An implicit solvent approach (Langevin dynamics) was used. The integration time step was set to d*t* = 0.01*τ*_Br_ (*τ*_Br_ = *k*_B_*T*/*Γ* is the Brownian time and *Γ* is the friction, set to 1 in Lennard-Jones (LJ) units) and the temperature to *T* = 1*ϵ*/*k*_B_ (*ϵ* is the characteristic interaction energy). Initial configurations were prepared by performing first an equilibration run for 5 × 10^5^*τ*_Br_ while attraction between DNAnss was not allowed. Then, we turned on the Morse attraction and performed a production run for 10^6^*τ*_Br_. We identified hybridization between adjacent nanostars when their patches were located at a distance *r* ≤ 0.2*σ* (the cut-off distance of the attraction between patches).

Elasticity was measured in simulations first by mimicking MR experiments in which we embedded a large bead in the sample and computed the ${G}_{{\mathrm{p}}}^{{\prime} }$ from the mean squared displacement of the bead (Supplementary Fig. 1). We also performed Green–Kubo simulations and found the elastic plateau from the autocorrelation of the stress tensor (Supplementary Section V and Supplementary Fig. 6).

## Online content

Any methods, additional references, Nature Portfolio reporting summaries, source data, extended data, supplementary information, acknowledgements, peer review information; details of author contributions and competing interests; and statements of data and code availability are available at 10.1038/s41563-024-02091-9.

## Supplementary information

Supplementary Information Supplementary Figs. 1–18, Tables 1–3 and Discussion.

## Data Availability

Datasets created during the current study are freely available via Edinburgh Datashare at 10.7488/ds/7839 (ref. ^55^). These data are also available via GitLab at https://git.ecdf.ed.ac.uk/ygutier2/data-j1Topo.

## Code availability

The codes used to perform the simulations and topology analysis and generate the figures are freely available via Edinburgh Datashare at 10.7488/ds/7839 (ref. ^55^) and via GitLab at https://git.ecdf.ed.ac.uk/ygutier2/j1Topo.
