# Supplementary material for: Topological linking determines elasticity in limited valence networks
Source: Nat Mater. 2025 Jan 31;24(3):454–61. doi: 10.1038/s41563-024-02091-9 (PMC11879876; doi:10.1038/s41563-024-02091-9)
Supplement: Supplementary file 1 — Supplementary Figs. 1–18, Tables 1–3 and Discussion. [file 41563_2024_2091_MOESM1_ESM.pdf]

nature materials

Supplementary information

https://doi.org/10.1038/s41563-024-02091-9

# Topological linking determines elasticity in limited valence networks

In the format provided by the authors and unedited

## CONTENTS


I. Nanostars Design 2

II. DNA hydrogels preparation 2

III. Protocols for rheology experiments 2
- Microrheology 2
- Oscillatory Bulk Rheology 3

IV. Details of simulations 5
- Equilibration and assemble of the network. 6
- Radial distribution function. 7
- Mean Squared Displacement 7

V. Elasticity from simulations 7
- Microrhelogy simulations 7
- Green-Kubo simulations 8

VI. The overlapping concentration 8

VII. Topology analysis 9
- Network diagrams 9
- Shortest path between branching points 10
- Minimum loops 11
- Linking number 12
- Average life-time of minimum loops 13

VIII. Interpenetration from experiments 14
- Intensity of the self-entangled and the two-species-entangled regions 16
- Temperature variations 16

IX. Results from simulations with a binary system of DNAns 18

X. Results with a non-rigid model of DNAns 18

References 19

## I. NANOSTARS DESIGN

The ease of design and realisation of DNAns and the ability to encode sequence-specific interactions make them ideal building blocks to achieve the bottom-up formulation of hydrogels with bespoke rheology [1, 2]. DNAns hydrogels are also being tested for a number of biotechnology applications, for instance spinal cord [3] and bone [4] regeneration, biobatteries [5], and sensors [6] and are key components of next generation artificial cells [7] and smart condensates [8].

In this work, nanostar motifs are assembled from three single-stranded (ss) oligonucleotides, 49 nucleotides long each and with sequences reported in Table S1. These motifs were designed using NUPACK [9], with minor modifications from those originally proposed in Ref. [10], and they consist of five functional parts. Each double-stranded (ds) DNA arm is 20 base-pairs (bp) long and is formed through the hybridization of segments I and II. The arms terminate in a self-complementary 6-nucleotide fragment with sequence 5'-CGATCG-3'. This sticky end is equal for all three arms, allowing the non-specific hybridization of two nanostars (ns): any of the three arms of one ns can hybridize with any (but only one) of the arms of another ns. Unpaired adenines (A) are introduced at the core of the Y-shaped structure and before the sticky end as flexible joints because they enhance the internal flexibility of the nanostar and the flexibility of the nanostar–nanostar bond, respectively.

Beyond structural features, the design provides certain stability. Using NUPACK and DINAmelt [11], we checked that at [NaCl]= 150mM and [ssDNA]= 500$\mu$M, the melting temperature of individual DNAns ($T_{m1} = 77°C$) is larger than the melting temperature of the sticky-ends ($T_{m2} = 38°C$). Therefore, for temperatures below $T_{m2}$ and at concentrations $C > 300\mu M$, beyond the phase-separation region [12], we can form DNAns hydrogels.

| | Segment I | FJC | Segment II | FJ | Sticky end | |
|---|---|---|---|---|---|---|
| 5′– | CTGGATCCGCGGAAGCTTAA | AA | CGGAATTCGCATGGATCCCC | A | CGATCG | –3′ |
| 5′– | GGGGATCCATGCGAATTCCG | AA | CTGAATTCCCTGGGATCCCG | A | CGATCG | –3′ |
| 5′– | CGGGATCCCAGGGAATTCAG | AA | TTAAGCTTCCGCGGATCCAG | A | CGATCG | –3′ |

Table S1. Strand sequence used in the nanostar design with valence $f = 3$. Each row represents a different ssDNA oligonucleotide. Each oligonucleotide is 49 bases long and presents two segments (20 nucleotides long each) separated by two unpaired A-nucleotides entailing flexibility at the molecule's core (FJC). Segments with the same colour have complementary sequences to form the double-stranded arms. The sticky end of each oligonucleotide is preceded by an unpaired A-nucleotide forming a second flexible joint (FJ).

## II. DNA HYDROGELS PREPARATION

Oligonucleotide sequences (see Table S1) were acquired already purified with a standard desalting process from Integrated DNA Technologies (IDT, https://www.idtdna.com/pages). The oligo stocks are delivered in a dehydrated state. Therefore, before opening the tubes, we centrifuged them for 15-30s to guarantee that all dried DNA was pulled down to the bottom. Afterwards, the stocks were resuspended in Ultrapure Water to 1mM, and we measured their concentrations employing an UV spectrophotometer (Thermo Scientific, NanoDrop Lite) after performing a series of dilutions in water at a ratio of 1:10, 1:100 and 1:1000. Once the concentration of each oligo was checked, we picked a volume from each stock for the three filaments to be equal in moles and mixed them into a single test tube. Then, we dehydrated the final solution at 60°C for roughly 2h in a vacuum concentrator (Eppendorf, Concentrator *plus*) by leaving the tube opened with a sterilised filter on the top to avoid contamination. Once completely dried, the sample was dissolved in the Nanostar buffer (40mM Tris, 40mM sodium acetate, 1mM EDTA (pH 8.0) and 150 or 500mM NaCl depending on the experiment) [13] by alternating heating at 60°C and vortexing. The amount of buffer used depends on the final concentration desired for the experiments. Finally, we performed the annealing step by heating the sample to 90°C for 2 min in a well-isolated heat block and then cooling it slowly down until 25°C was reached in the block (over 4 h). Before performing any microrheology and confocal experiments, we measured the absorption at 260 nm (A260) using the same NanoDrop after 1:100 (with absorbance $A260_{1\%}$) and 1:1000 (with absorbance $A260_{0.1\%}$) dilution in water and following the calculations suggested in Ref. [13] (assuming the absorbance of the 0.1% solution is due to only ssDNA). Our results are summarised in Table S2 and they suggest that $\alpha$ ranged from 17% to 43%, while correction to the concentration (errors) is in the range 17% to 28%. Finally, the DNA Nanostar solutions were stored at 4°C.

## III. PROTOCOLS FOR RHEOLOGY EXPERIMENTS

### Microrheology

In this section, we use particle tracking microrheology (PTM) to characterise the rheological properties of DNA hydrogels at different concentrations of DNA nanostars. In this technique, the stress relaxation moduli of a fluid are obtained from the mean squared displacement of spherical probes embedded in it. We report the results obtained using PTM in both experiments and simulations.

| Concentration [$\mu$M] | $A260_{1\%}$ | $A260_{0.1\%}$ | $\alpha$ | Correction |
|---|---|---|---|---|
| 200 | 2.35 | 0.28 | 0.38 | 1.19 |
| 250 | 2.92 | 0.35 | 0.36 | 1.20 |
| 300 | 3.45 | 0.42 | 0.31 | 1.22 |
| 320 | 3.80 | 0.45 | 0.40 | 1.18 |
| 380 | 4.51 | 0.53 | 0.43 | 1.18 |
| 400 | 4.39 | 0.56 | 0.17 | 1.28 |
| 450 | 5.28 | 0.63 | 0.38 | 1.19 |
| 470 | 5.42 | 0.66 | 0.31 | 1.22 |
| 500 | 5.87 | 0.69 | 0.43 | 1.17 |
| 520 | 6.14 | 0.73 | 0.39 | 1.19 |
| 550 | 6.02 | 0.76 | 0.20 | 1.26 |
| 560 | 6.54 | 0.78 | 0.38 | 1.19 |

Table S2. **Concentration measurements in MR samples**. First column is the concentration of DNAns samples. Next two columns show results of the absorbance at 1:100 and 1:1000 dilutions. Fourth column is the calculated percentage of single-stranded DNA present at 1%: $\alpha = (\frac{A260_{1\%}}{A260_{0.1\%}} - 7.4)/2.6$ with range in the interval $\alpha \in [17\%, 43\%]$. Last column contains the correction to the concentration of DNAns at 1%: $A260_{ss}/A260_{1\%} = 1/(0.26\alpha + 0.74)$, with the correction ranging in the interval $[17\%, 28\%]$.

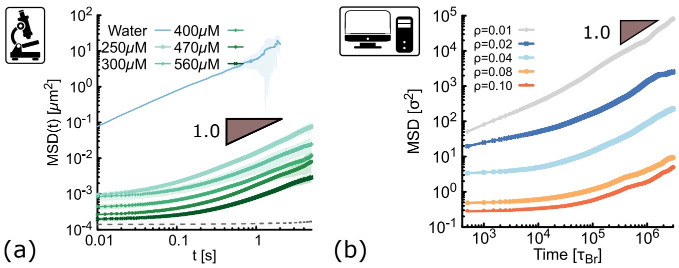


Figure S1. **Microrheology.** **(a)** Examples of $MSD(t)$ obtained from experiments via PTM at different DNAns concentration. To check sample homogeneity and obtain reliable statistics, each MSD at a fixed concentration (1 replicate) represents the mean of MSDs computed over at least 9 different positions within the same sample, with error bars indicating the standard deviation. The grey dashed line shows the MSD of beads immobilised on the glass surface. **(b)** shows analogous results of the $MSD(t)$ obtained from MR simulations (details of the model in sections IV and V). By using the Generalised Stokes-Einstein Relation, the elastic ($G'(\omega)$) and viscous ($G''(\omega)$) modulus as function of the frequency reported in the main text, Fig. **1**(c) (for experiments) and Fig. **1**(g) (for simulations), are obtained.

Before starting the microrheology (MR) experiments, solutions at different concentrations of DNAns were prepared. Here we describe the preparation of a sample at a final concentration [DNAns]= 500$\mu$M; the generalisation of this protocol for different concentrations is straightforward. A total volume of 10$\mu$L is used in all our microrheology experiments. We start by incubating an Eppendorf tube in a heat block set at 60°C and add: (i) 5$\mu$L of DNAns at 1mM (49$\mu$g/$\mu$L) pre-heated at 60°C for 2 minutes. (ii) 4.5$\mu$L of Nanostar Buffer (150mM NaCl, 1mM EDTA, 40mM Tris, 40mM Acetic Acid) to keep the salt concentration constant. (iii) 0.5$\mu$L of 200 nm polystyrene beads (Sigma-Aldrich, d=1.05g/cm$^3$, concentration 10% solid), previously diluted in 1:500 in water. Once the beads are spiked in the solution, the samples are well mixed by pipetting 3-5 times via tips, which are also heated at 60°C to prevent quick cooling of the sample inside the tips and help the tracer particles' homogenisation into the self-assembling gel.

Samples are sandwiched between a glass slide and a cover slip to avoid drift and evaporation using a $\sim$ 100$\mu$m sticky spacer. The samples are left to equilibrate for a couple of minutes to reach 25°C in a stage-top temperature-controlled chamber (OKO Lab), which is placed on an inverted Nikon microscope and imaged with a 100x oil immersion objective in bright field mode. We restrict the field of view to 512x512 pixels and track 5-10 beads at 400 fps for 5 seconds. Recall that the typical bond lifetime between two sticky ends is roughly 0.1 s at 25°C [13]. To extract the particles' trajectories, we use trackpy (`github.com/soft-matter/trackpy`) and compute their mean squared displacement (MSD). To ensure good statistics, we tracked the same amount of beads in different positions of the sample. Figure S1(a) shows the results obtained. We note that all our MR data is provided at concentrations $C < c^* = 600\mu$M, this is due to a technical limitation in the microscope's camera resolution, which we evaluated by analysing the Mean Squared Displacement from beads adhered to the glass surface (grey dashed-line in Fig. S1(a)). This line indicates the camera's limit to distinguish significant movement of the beads from background noise. Since the MSD obtained for samples at $C = 560\mu$M only marginally exceeds this limit, we then refrained from exploring higher concentrations.

To further characterise the viscoelastic properties of the system, we use the generalised Stokes-Einstein relation (GSER) to compute the complex stress modulus [14, 15]. Results of the viscous and elastic modulus as a function of the frequency are reported in Fig. **1**(c) of the main text. Experiments were repeated from the preparation step 2-3 times for each concentration (see Table S2). The results from all the experiments are reported in Fig. S2(a) below. There, we show the scaling of the high-frequency elastic plateau ($G'_p$) with the concentration. The fit to a power law to this data (at $C > 300\mu$M, beyond the phase-separation region), returns $G'_p \sim C^{2.5\pm0.1}$.

## Oscillatory Bulk Rheology

We employed bulk rheology (BR) to characterise the rheological properties of DNA hydrogels at different con-

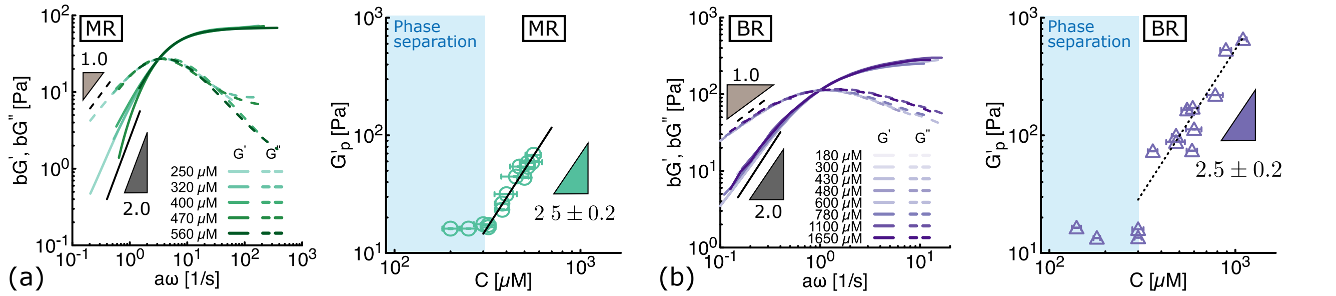


Figure S2. **Elasticity from rheology experiments. (a),(b)** Left panels show post-superposition frequency curves for microrheology (shifted relative to the 560$\mu$M curve) and bulk rheology (shifted relative to the 600$\mu$M curve) experiments, for a variety of DNAns concentrations (pre-superimposed data is shown in Fig. **1**(c),(d) of the main text). Right panels show the scaling of $G'_p$ and $G_0$, from MR and BR experiments, respectively. The blue shadow area represents concentrations at which the system phase separates [12]. For MR, samples are obtained from three independent 1mM DNAns batches (following the protocol in Section II) and diluted as needed. For BR, each data point is an independent sample prepared at the desired concentration for each measurement. In both MR and BR, the errors shown along the x-axis are technical, as it was challenging to replicate exact concentrations across samples. Therefore, we present a single $G'_p$ and $G_0$ value measured from each sample, with error bars for DNAns concentration calculated following the method in reference [13] (see Table S2). In Fig. **1**(e) of the main text we show the same data but without errors in concentration to ease visualization. We note that the data from MR was obtained at T=25°C, while from BR at T=20°C.

centrations of nanostars. We utilise a stress-controlled Kinexus Pro rheometer by NETZSCH. In an identical manner to the samples made for MR, DNAns solutions at different concentrations were prepared (except that polystyrene beads were not added).

The DNAns solutions were pre-heated at 60°C for 2 minutes, then 100$\mu$l was pipetted, using pre-heated tips, onto a flat 40 mm bottom plate. The top 40 mm plate was quickly closed to a 200 $\mu$m gap, the excess sample trimmed, and a solvent trap quickly fitted. The solvent trap uses mineral oil to create a temperature-stable seal and some wetted paper towel scraps within the chamber for rapid humidity equilibration. The sample is then reheated to 60°C to re-anneal it before measurements are performed. We also attempted to replicate an oil seal as used by Conrad [13] but a mixing of the oil with the sample was observed post-measurements. This may be due to the temperature increase in this re-anneal step which lowers the surface tension of the oil.

For each concentration, an amplitude sweep was initially performed at a temperature of 20°C, frequency of 10 Hz, and a shear strain range of $\gamma = 0.1\% - 10\%$ to determine the linear viscoelastic region (LVR).

Frequency sweeps were subsequently performed at a shear strain of $\gamma = 0.5\%$ within the LVR. DNAns solutions behave like Maxwellian viscoelastic fluids, with (i) a low frequency liquid behaviour ($G'' > G'$, $G' \propto \omega^2$, and $G'' \propto \omega$), (ii) a cross-over frequency ($\omega_0$) and (iii) a high-frequency solid-like behavior ($G' > G''$), and with a plateau modulus $G'_p$ (see Fig. **1**(d) of the main text and Fig. S2(b)). This behaviour is in agreement with previous experiments [13] and is also due to the reversible nature of the hybridisation between DNAns, which has a characteristic timescale $\sim \omega_0^{-1}$.

After measurements were performed, DNAns were collected, and the concentration of DNA was remeasured. In all samples, the concentration of DNA post-measurement was found to have increased. This post-BR concentration was noted as the concentration of the sample.

From the frequency sweeps, it was possible to superimposed $G'$ and $G''$ from different concentrations into a master curve. This was done by first finding the crossover frequency ($\omega_0$) and modulus ($G_0$), at which $G' = G''$. Then we rescaled frequencies (by a constant factor $a = \omega_{0,ref}/\omega_0$), and the elastic and viscous modulus (by the constant factor $b = G'(\omega_{0,ref})/G_0$). Here, $\omega_{0,ref}$ and $G'(\omega_{0,ref})$ are the reference values obtained from results at [DNAns]$= 600\mu M$. Fig. S3(a) shows the dependence on the rescaling factors as function of the concentration. Left panel in Fig. S2(b) shows results from BR after rescaling. We note that all the frequency curves overlap well above $300\mu M$. Below this point, at $C < 300\mu M$, they actually overlap before curves are superimposed. Above 1mM, a slight deviation in $G''$ exists at high frequencies. Putting them in the Maxwell model's context is likely irrelevant as they are a viscous contribution on a time scale associated with solid behaviour.

In terms of determining how the plateau modulus $G'_p$ scales with concentration, it is equivalent to tracking how $G_0$ scales with concentration, and $G_0$ is significantly easier to track being a well-defined point. This is displayed in the right panel of Fig. S2(b) and also reported in Fig. **1**(d) of the main text.

Amplitude sweeps performed at T=20°C and 1 Hz are reported in the left panel of Fig. S3(b). At low strains, all samples exhibit linear elasticity for which $G'$ is constant. At high strains, there is a critical value $\gamma_m$ at

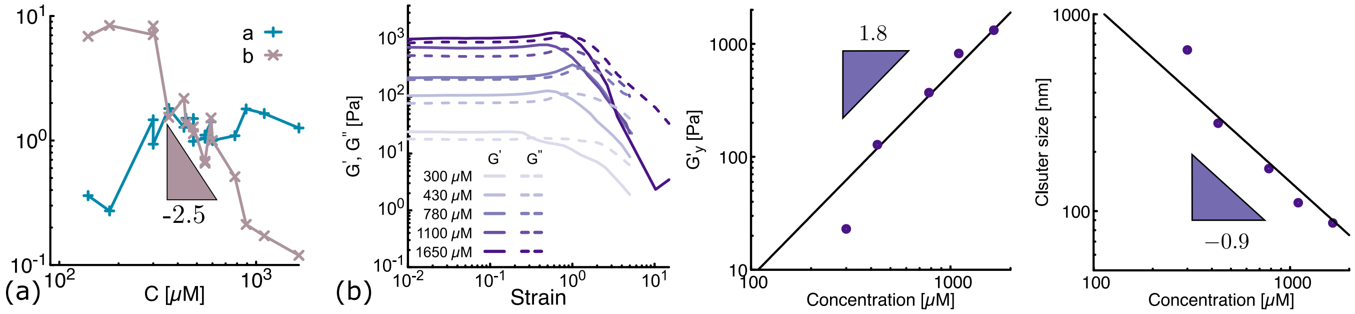


Figure S3. **Further analysis from BR at T=20°C.** **(a)** Rescaling factors ($a = \omega_{0,ref}/\omega_0$ and $b = G'(\omega_{0,ref})/G_0$) as function of the DNAns concentration. These values were applied to the BR measurements in Fig. **1**(d) of the main text in order to obtain the left panel in Fig. S2(b). Since $b \sim 1/G_0$, this factor has a scaling behaviour inverse to the one of the elastic plateau. On the other hand, the $a$-factor is roughly constant. **(b)** From left to right we show the strain sweep measurements at different concentration of DNAns, the strain hardening and the inferred cluster size. The solid lines are guides for the eye.

which $G'(\gamma_m)$ shows a sudden decrease or yielding of the network, typically associated with bond breaking.

In the middle panel of Fig. S3(b) we show the yield stress ($G'_y$) as function of the DNAns concentration. This exhibits a power law behaviour $G'_y \sim C^{1.8}$. In agreement with [13], our results confirm the scaling of the inferred cluster size with the concentration of DNAns (see right panel of Fig. S3(b)), where we used the relation in which the cluster size is proportional to $(F_y/G'_y)^{1/2} \sim C^{-0.9}$, with $F_y = 10$ pN the characteristic bond-breaking force for a 6 base-pairs overhang.

In the discussion so far, MR was performed at room temperature, stabilised at T=25°C in a thermal chamber, while BR was performed at T=20°C. For completeness and better comparison, here we report results from BR at T=25°C (see Fig. S4). We found that: (i) the value of the elastic plateau is not greatly affected by this change in temperature, and as expected, $G'_p$ is in general smaller at higher temperatures. (ii) The relaxation time ($\tau_c = 1/\omega_0$) obtained from the cross-over frequency ($\omega_0$) decreases from $\tau_c \sim 1-2$ seconds at T=20°C, to $\tau_c \sim 0.2-0.4$ seconds at T=25°C, in agreement with [13]. (iii) The scaling exponent of the elasticity with concentration of DNAns is not affected by the change in temperature. Therefore, the fact that the $G'_p$ measured from MR is systematically smaller than the one measured in BR is likely due to the interaction between the DNA and the surface of the probe particle, as discussed in a recent paper [16].

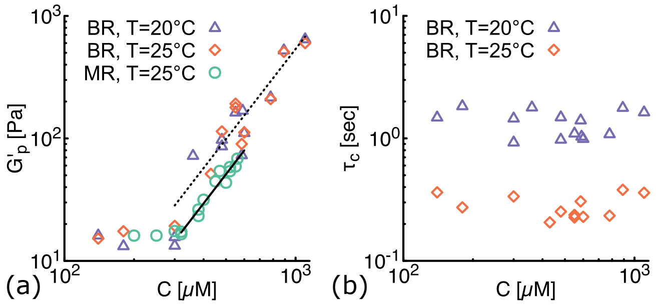


Figure S4. **Results from rheology experiments at T=20°C and T=25°C.** **(a)** Scaling of elasticity with concentration of DNAns. **(b)** Relaxation time as function of $C$.

## IV. DETAILS OF SIMULATIONS

As described in the main text, we model DNA nanostars as rigid bodies made up of ten particles. Seven beads constitute the core of the molecule. Each bead has a diameter $\sigma \sim 2.5$ nm (or 8 bp) implemented via a truncated and shifted Lennard-Jones (LJ) potential:

$$U_{LJ}(r) = 4\epsilon\left[\left(\frac{\sigma}{r}\right)^{12} - \left(\frac{\sigma}{r}\right)^{6} + \frac{1}{4}\right], \qquad \text{(S1)}$$

if $r < 2^{1/6}\sigma$, and $U_{LJ}(r) = 0$ otherwise. Here $\epsilon = 1.0$ parameterises the strength of the repulsion, and $r$ is the Euclidean distance between the beads.

Patches are placed at a distance of $2.5\sigma \sim 20$bp from the core of the molecule and on the surface of the outermost bead along each arm (see Fig. **1**(f) in the main text). To represent the sticky-ends interaction and to regulate the attraction between adjacent nanostars, we use a Morse potential:

$$U_m(r) = \epsilon_m\left[e^{-2\alpha_0(r-r_0)} - 2e^{-\alpha_0(r-r_0)}\right], \qquad \text{(S2)}$$

for $r < R_c$. Here, $r$ represents the distance between patches of two nanostars, $r_0 = 0$ is their equilibrium distance, and $R_c = 0.2\sigma$ is the cut-off distance of attraction. We set $\epsilon_m = 25.0k_BT$ and $\alpha_0 = 14\sigma^{-1}$ to control the amplitude and width of the potential, respectively. However, our nanostars design constraints the minimum distance between patches to be $0.12\sigma$ and therefore, the effective energy of hybridization is about $10k_BT$ (see [17] for details). With these parameters the single-bond-per-patch condition holds in the bast majority of our results.

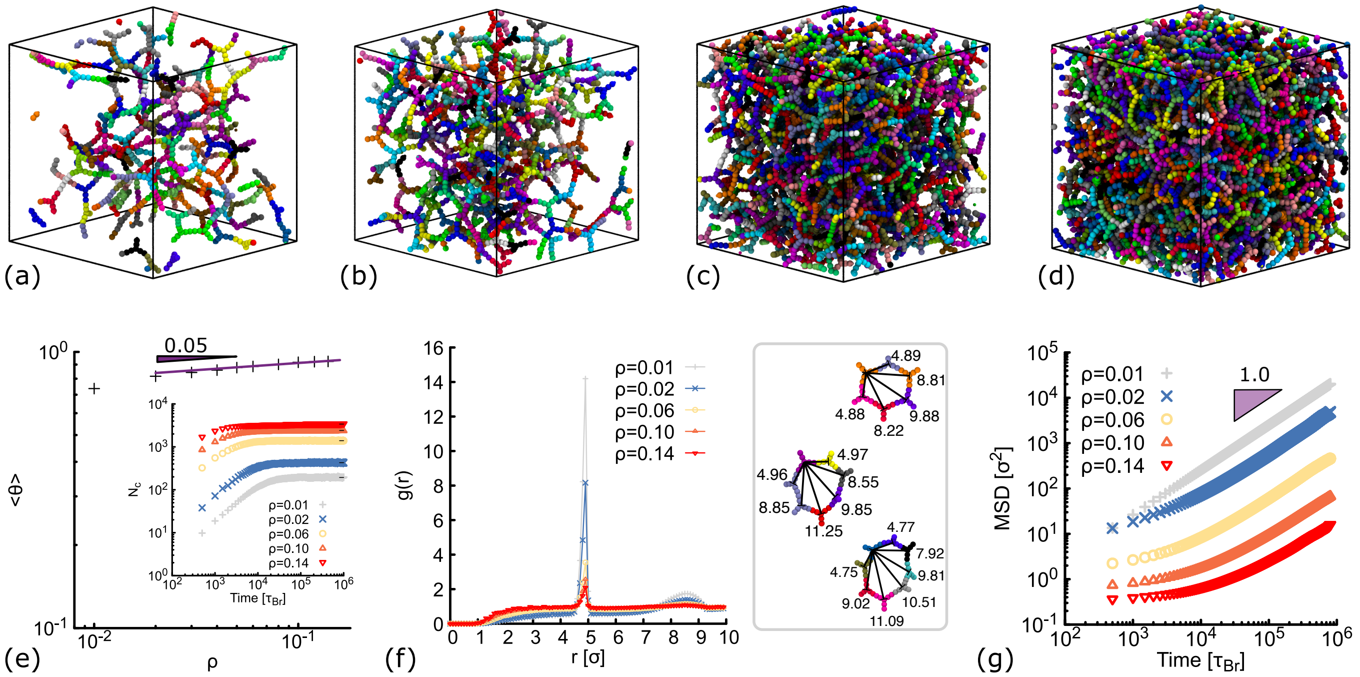


Figure S5. **Network assemble**. Panels **(a)**-**(d)** show snapshots of typical configurations from simulations after the network is formed, at the last time-step ($t = 10^6\tau_{Br}$) and at $\rho = 0.01, 0.02, 0.06$ and $0.1$, respectively. **(e)** The curves in the inset represent the number of contacts $N_c$ as function of time obtained during the network formation. The main plot shows the fraction of connected nanostars in the steady state as a function of the volume fraction. **(f)** In the left panel, the radial distribution function averaged over configurations at long times (when the network is formed) is shown. The right panel shows typical geometries found in simulations at $\rho = 0.01$. Black lines connect the core of two DNAns, and the labels show the distance (in simulation units) between them. **(g)** Log-log plots of the MSD averaged over all the molecules in the system.

At a given concentration, on average only 0.002%-0.025% of all the contacts in simulations with our model would not satisfy the one-to-one binding. Importantly, the hybridisation between DNAns is not permanent; there is a characteristic time ($\tau_c$) for network reconfiguration in which nanostars unbind and bind again to a different ns.

The Langevin integration of the system was carried out using LAMMPS [18] in an NVT ensemble by a standard velocity-Verlet algorithm with integration time-step $dt = 0.01$. The position of particles in the system obeys the following equation:

$$m\frac{d^2\mathbf{r}}{dt^2} = -\mu\frac{d\mathbf{r}}{dt} - \nabla U + \sqrt{2k_BT\mu}\Lambda(t)\,, \qquad \text{(S3)}$$

where $U$ is the total potential field experience by a particle, $m = 1$ is the mass of the particle, $\mathbf{r}$ represents its position, $\mu$ is the friction and $\Lambda(t)$ is the white noise term with zero mean which satisfies $\langle\Lambda_\alpha(t)\Lambda_\beta(s)\rangle = \delta_{\alpha\beta}\delta(s-t)$ along each Cartesian coordinate represented by the Greek letters.

### Equilibration and assemble of the network.

Initial configurations were produced by placing $N$ nanostars in a cubic simulation box of length $L = 40\sigma$ and with periodic boundary conditions (PBC). The volume fraction of this system is $\rho = NV_1/L^3$, where $V_1 = 7\frac{4}{3}\pi(\sigma/2)^3$ is the excluded volume of one nanostar, given by the sum of the volume of all its structural beads. During equilibration, we turn-off the attraction between nanostars ($\epsilon_m = 0$) and integrate the system for $5 \times 10^5\tau_{Br}$. We then set the attraction between patches to $\epsilon_m = 25k_BT$, allowing in this way the network formation, and we run the system for $10^6\tau_{Br}$. Snapshots from simulations at the end of this run are depicted in Figs. S5(a)-(d) for different DNAns concentrations.

We compute the total number of contacts ($N_c(t)$) between DNAns at a fixed time-step by counting the number of pairs of patches that are at a distance $r \leq 0.2\sigma$. The temporal evolution of this quantity is reported in the inset of Fig. S5(e). At all concentrations, the system evolves to a steady state where the network is formed and $N_c(t)$ plateaus around an equilibrium value. The fraction of connected DNAns ($\langle\theta\rangle = 2N_c(t)/Nf$) in this steady state is obtained by averaging the value of $N_c$ at $t \geq 2\times10^5\tau_{Br}$. The plot of $\langle\theta\rangle$ as a function of the volume fraction is reported in Fig. S5(e), from which we can extract the scaling $\langle\theta\rangle \sim \rho^{0.05}$, i.e. very weakly dependent on the volume fraction. Indeed, for most of the values of $\rho$ explored in this work, the fraction of connected DNAns

is always near unity.

### Radial distribution function.

As an initial attempt to characterise the structure of our networks, we compute the radial distribution function (RDF), g(r), using only the position of beads at the core of the DNAns:

$$g(r) = \frac{1}{4\pi\phi M}\sum_{i=1}^{M}\sum_{j\neq i}^{M}\langle\delta(|r_{ij}-r|)\rangle, \qquad \text{(S4)}$$

where $\phi = N/L^3$ is the number density of core beads in the simulation, $r_{ij}$ represents the distance between the cores of molecules $i$ and $j$, and the sum counts the number of DNAns cores that are at a distance $r$. We compute $g(r)$ using the wrapped coordinates of nanostars in our simulations by: (i) Choosing the $M$ core beads that are inside a sphere of radius $R_{select} = L/4$ with centre at the origin of the box. (ii) We loop over each of these $M$ particles and count the number of core beads neighbours at a distance $r$. This search radius is always smaller or equal to $L/4$, and, in the count, we consider all the core beads in the simulation (not only the $M$ selected in (i)). (iii) We replace this information in Eq. S4 to compute $g(r)$. Then, we repeat this procedure for all configurations from simulations in the steady state (at large times and when $\theta(t)$ has reached a plateau), and we take the average over configurations. Results at different concentrations are depicted in the left panel of Fig. S5(f). At all values of $\rho$, $g(r)$ displays a global maximum located close to $r = 5\sigma$. This corresponds to the average distance between the core of two bound nanostars. We note that at low concentrations, for instance at $\rho = 0.01$, a peak also appears at $7.5\sigma < r < 9\sigma$, with a local maximum at about $r = 8.5\sigma$, corresponding to the distance between second nearest neighbours. Loops made by nanostars observed in simulations are consistent with these results (see the right panel of Fig. S5(f)). As the nanostar concentration increases, the global maximum height decreases, and the second peak flattens, indicating a more heterogeneous, disordered network with broader distributions of structures and network loops.

### Mean Squared Displacement

We compute the mean squared displacement of the centre of mass of nanostars after network formation using the relation:

$$\text{MSD}(t) = \langle[\boldsymbol{r}_{CM}(t+t_0) - \boldsymbol{r}_{CM}(t_0)]^2\rangle, \qquad \text{(S5)}$$

where the average is performed over nanostars and $t_0$. We report the MSD at different concentrations in Fig. S5(g). As expected, the system's mobility decreases as the nanostars concentration increases. We also note that while at $\rho = 0.01$, the MSD shows a diffusive behaviour, for larger values of $\rho$, the MSD displays a sub-diffusive regime at early times, which ends in a freely diffusive regime at late times. This is a typical signature of viscoelastic behaviour that is also captured by the plateau of $G(t)$ in Fig. **1**(h) of the main text.

## V. ELASTICITY FROM SIMULATIONS

### Microrhelogy simulations

We simulate PTM experiments by introducing a $15\sigma$ diameter probe-particle (larger than the mesh size, see Fig. **2**(c) of the main text) in our simulation box. The excluded volume interaction of this particle is implemented via a purely-repulsive LJ potential, similar to the one in Eq. S1. The difference in particle size between the probe and nanostar beads can lead to a computationally inefficient integration of our system. Therefore, we use the *multi-style* of the neighbour command implemented in LAMMPS [18]. Each nanostar bead is subject to a translational drag $\mu$ that can be related to an inertial time scale $\tau_{in} = m/\mu$, i.e., the time over which the velocity decorrelates (directional information is lost). For a sphere, Stokes' law relates the drag to the viscosity of the solvent $\eta$: $\mu = 3\pi\eta d$, where $d$ is the diameter of the sphere. Therefore, the inertial time of a nanostar bead ($\tau_{in} = m/3\pi\eta\sigma = 1.0$) is set 15 times larger than that of the probe ($\tau_{in,p} = \tau_{in}/15 = 0.06$).

Once the probe particle is embedded into the solution of nanostars, we perform an equilibration run for $10^6\tau_{Br}$ time-steps, during which we prevent the attraction of nanostars ($\epsilon_m = 0$). Then, we reset the timestep to zero, turn on the attraction between nanostars and run the system for $5 \times 10^6\tau_{Br}$. The mean squared displacement of the probe-particle is reported in Fig. S1(b) for simulations at different concentrations of DNAns. As expected, the higher the concentration of nanostars, the slower the dynamics of the bead. Just as in experiments, we used the GSER to compute $G'(\omega)$ and $G''(\omega)$, reported in Fig. **1**(g) of the main text. The elastic plateau $G'_p$ in Fig. **1**(i) is extracted as the value of $G'$ at the largest frequency ($\omega \sim 2 \times 10^{-3}\tau_{Br}$). We note that since simulating MR experiments is computational expensive, we performed only one replica per DNAns concentration and the red points reported in Fig. **1**(i) do not include error-bars. However, the scaling $G'_p \sim \rho^{2.5}$ is in agreement with the results found in experiments.

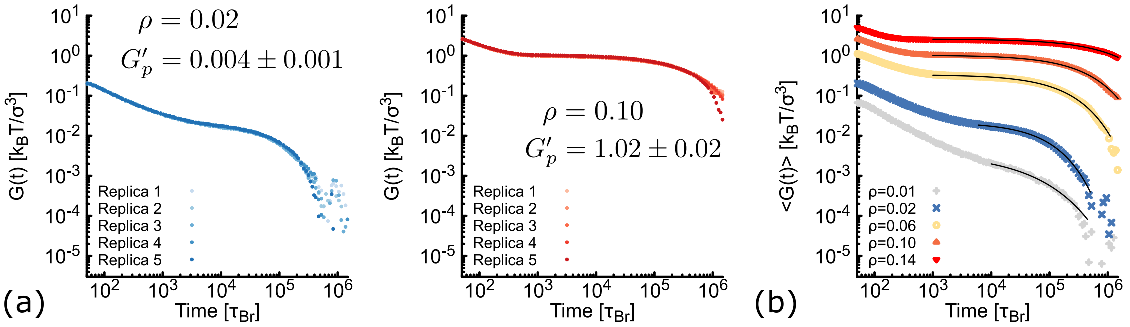


Figure S6. **Results from Green-Kubo simulations.** Autocorrelation of the stress-tensor obtained from equilibrium Green-Kubo simulations. **(a)** Shows results from five independent replicas at two different concentrations, $\rho = 0.02$ (left) and $\rho = 0.1$ (right). **(b)** Average over replicas of the stress relaxation function at different concentration of nanostars. Points represent simulation results and black lines are the fits of a stretched exponential function. Labels in panel (a) give the elastic plateau and error obtained by this fitting. The curves are also well fitted by a Maxwellian behaviour with two modes.

### Green-Kubo simulations

The time-dependent mechanical response of a viscoelastic material in the limit of zero-deformation can be computed from the autocorrelation of the pressure tensor at equilibrium [19]:

$$G(t) = \frac{L^3}{3k_BT}\sum_{\alpha\neq\beta} P_{\alpha\beta}(0)P_{\alpha\beta}(t), \quad \text{(S6)}$$

where $P_{\alpha\beta}$ represents the out-off diagonal component ($P_{xy}$, $P_{xz}$ and $P_{yz}$) of the stress tensor. The autocorrelation was computed using the multiple-tau correlator method described in reference [20] and implemented in LAMMPS with the *fix ave/correlate/long* command. This computation ensures that the systematic error of the multiple-tau correlator is always below the level of the statistical error of a typical simulation.

At each concentration of nanostars we ran five independent replicas. Results from long simulations ($5\times10^6\tau_{Br}$) at $\rho = 0.02$ and $\rho = 0.1$ are shown in Fig. S6(a). The average over replicas is then reported in Fig. S6(b). We observe that the autocorrelation function exhibits a power law decay at early times, followed by an intermediate plateau and ending with structural relaxation. This is a typical signature of a viscoelastic fluid [21]. The final decay of $G(t)$ is well fitted by a stretched exponential function, $G'_p\exp[-(t/\tau)^\beta]$ (solid lines in Fig. S6(b)), where $\tau$ represents the relaxation time (see Fig. S12(b)). Using this information we obtained the scaling of the elastic plateau $G'_p \sim \rho^{2.5\pm0.1}$ reported in Fig **1**(i) of the main text. Finally, we have checked that the data in Fig. S6(b) is also well fitted by the sum of two exponential decays (Maxwellian behaviour with two modes) and with that fit we retrieve the same values of $G'_p$.

## VI. THE OVERLAPPING CONCENTRATION

The overlapping concentration ($c^*$) marks the point at which a solution is no longer dilute and polymer coils (or in this case nanostars), start to overlap. In experiments, $c^*$ is obtained [22] as:

$$c^* = \frac{M_w}{\frac{4}{3}\pi R^3 N_A}, \quad \text{(S7)}$$

where $N_A$ is Avogadro's constant, $M_w$ is the molecular weight of a single DNAns and $R$ represents the radius of gyration of a nanostar that is part of the network. Since each DNAns is made of 150 nucleotides, $M_w = 150 \times 650\text{Da}/2 = 48750g/\text{mol}$. The value of the radius of gyration is estimated by assuming that the double-stranded (ds) arms of a DNAns are rigid straight segments with a length of 20 base-pairs (bp). This approximation is possible since the persistence length of ds-DNA (150 bp at [NaCl]=0.15mM) is larger than the arms themselves. When the DNAns is part of the network, the sticky-end contributes with an extra 6 bp length to each arm. Considering that the average height of a base-pair is $h = 0.34$ nm, we obtain $R = 8.84$ nm and therefore $c^* = 28.0$ mg/ml, or equivalently $c^* = 0.6$ mM.

In simulations, we expect that as we reach the overlapping concentration, the density of beads in the system ($7N/L^3$) is the same as the density of beads of a single DNAns ($7/V_{ns}$), where $V_{ns} = \frac{4}{3}\pi R^3$ is the volume occupied by a single DNAns with radius of gyration $R = 2.5\sigma$. Therefore $\frac{4}{3}\pi N^*(\frac{R}{L})^3 = 1$, from which we obtain the number of nanostars ($N^* = 978$) and the overlapping volume fraction $\rho^* = N^*(7\frac{4}{3}\pi\sigma^3)/L^3 = 0.056$. Our simulations suggest that at $\rho^*$ topological complex motifs like linking appear in the network, and therefore, $\rho^*$ separates two regimes in the scaling of the mesh size (Fig. **2**(c)) and the average linking number per minimum loop (Fig. **4**(g)). More specifically, at $\rho < \rho^*$, when the average linking between loops is negligible, we argue that

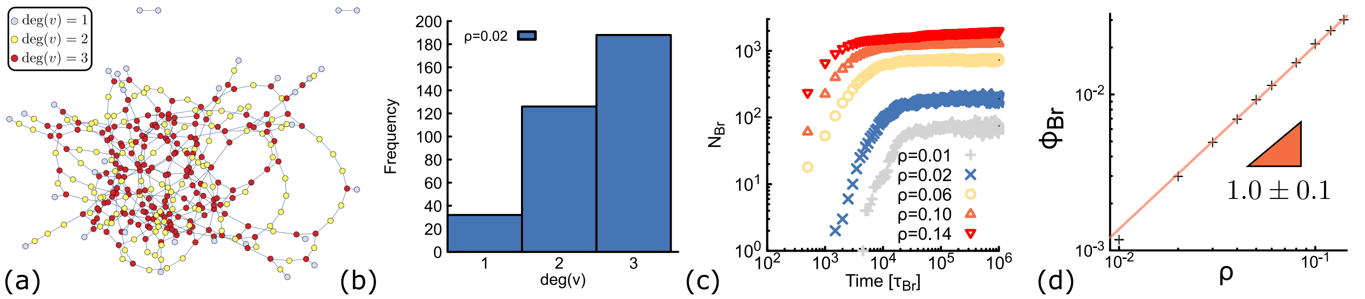


Figure S7. **Network diagram**. **(a)** Shows the network diagram corresponding to the system depicted in Fig. S5(b), at the last time-step ($t = 10^6\tau_{Br}$) of the simulation and at $\rho = 0.02$. **(b)** Histogram obtained from the network diagram in (a), depicting the frequency of each deg($v$). **(c)** Curves represent the number of branching points ($N_{Br}$) as a function of time obtained during the network formation. Using at least 25 independent configurations at long times, we compute the mean and SEM of $N_{Br}$ in **(d)**, where we report the scaling of the fraction of branching points with the volume fraction. Error bars are smaller than symbols.

the branching points and mesh size are the main determinants of the elasticity:

$$G'_{p,branch} \sim k_B T \frac{\phi_{Br}}{\xi^3} \sim \rho^{2.8} \tag{S8}$$

where we used that $\phi_{Br} \sim \rho$ and $\xi \sim \rho^{-0.6}$. This scaling is in relatively good agreement with our rheology measurements and simulations (see Fig. **4**(h), yellow line at $\rho < \rho^*$). The origin of Eq. S8 is phenomenological. As in classic theories [22], we argue that the elasticity of the system is directly proportional to the density of active crosslinks $\phi_{Br}$ but is also affected by the relevant length-scale, which for elasticity is the pore size ($\xi$). Since elasticity has units of energy over volume we obtain the relation in Eq. S8. It is also worth mentioning that since $N_{minloop}$ and $\phi_{Br}$ have the same scaling behaviour with $\rho$, another way to express the elasticity of the network is that $G'_p \sim N_{minloop}/\xi^3$. On the other hand, for $\rho \geq \rho^*$, we have shown in the main text that Eq. S8 leads to $G'_{p,branch} \sim \rho^{3.6}$ (see Fig. **4**(h), yellow line at $\rho \geq \rho^*$).

## VII. TOPOLOGY ANALYSIS

### Network diagrams

A network diagram is a representation of the connection between nanostars at a given time-step in the simulation. We identify these connections by computing the distance between the $3 \times 3 = 9$ pair of patches belonging to two different nanostars. If any of the results is smaller than $R_c = 0.2\sigma$, the two nanostars involved are connected. This information is visually captured in a network diagram by drawing vertices representing nanostars and a line (also called edge) between any two connected nanostars. In Fig. S7(a), we show the network diagram for one of the snapshots from simulations at $\rho = 0.02$. Different colours represent the degree of a vertex (deg($v$)), i.e., the number of DNA nanostars connected to that vertex. Since the valence of DNAns is $f = 3$, deg($v$) can be either: 0 (for isolated stars, not shown in the plot), 1 (light-blue circles), 2 (yellow circles) or 3 (red circles). The histogram depicting the frequency of nanostars with a certain degree of connection is also shown in Fig. S7(b). As it can be seen, the number of branching points (fully connected DNAns, with deg($v$) = 3) is larger than those of DNAns with deg($v$) = 1 or 2. This is reflected in the higher density of red circles in the network diagram of Fig. S7(a). By repeating this process at different time-steps, we obtain the temporal evolution of the number of branching points ($N_{Br}(t)$) reported in Fig. S7(c). We observe that at all concentrations $N_{Br}(t)$ reaches a plateau at long times, from which we compute the average and standard error

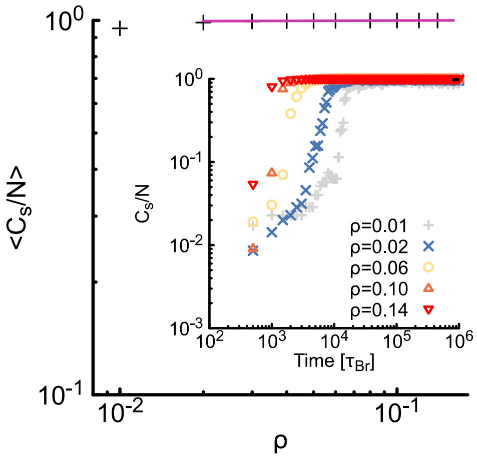


Figure S8. **Largest connected component**. Inset shows the time evolution of the number ($C_s$) of DNAns in the largest connected component, normalized by the number ($N$) of nanostars in the system. The main plot displays the scaling of $C_s/N$ in the steady state with the concentration of DNAns.

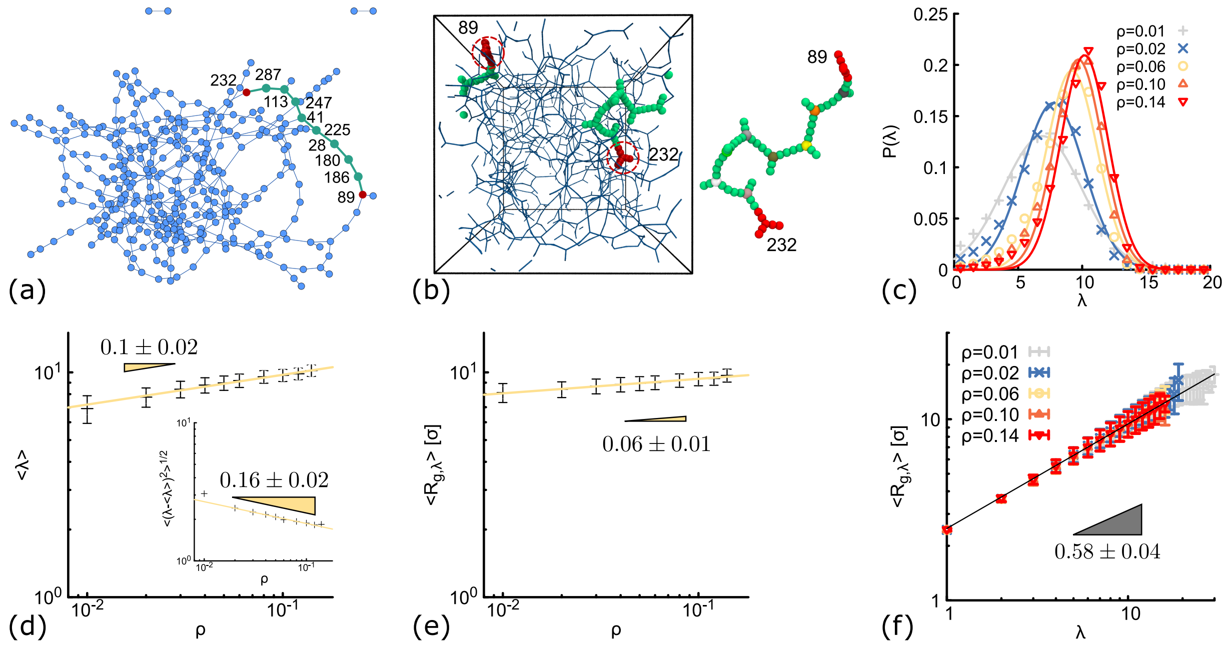


Figure S9. **The shortest path analysis**. **(a)** Shows the same network diagram as the one in Fig. S7(a), but with two branching points highlighted in red and the shortest path between them highlighted in green. Labels represent the molecule ID of nanostars. Note that the length of the shortest path is $\lambda = 9$ nanostars. **(b)** Left panel shows a snapshot from simulations corresponding to the network diagram in (a). Since the shortest path is formed through the periodic boundary conditions, we use the minimum image criterion to reconstruct the correct path (Right panel). To ease visualization, the core beads of nanostars are highlighted. **(c)** Probability distribution of the shortest path. Lines are fits to the data using a Gaussian function. **(d)** Scaling of $\lambda$ with the volume fraction of nanostars. Inset shows the variance of the probability distribution of the shortest path as function of $\rho$. **(e)** Scaling of the average radius of gyration of the shortest path as a function of $\rho$. **(f)** Plot of the radius of gyration (computed using Eq. S9) as function of the length $\lambda$, for different concentrations of nanostars. In panels (d)-(f) error-bars report the SEM which is obtained using measurements from five independent gel configurations at each volume fraction.

of the mean (SEM) of the number of branching points. This information is then used to produce Fig. S7(d) in which we report the number density of branching points ($\phi_{Br} = N_{Br}/L^3$) as function of the concentration of DNAns.

In network diagrams, a connected component is a set of vertices with edges spanning paths to connect any two of them. The larger the set of vertices in a component, the higher the degree of connectivity in the system. By inspecting the network diagram in Fig. S7(a), it is evident that most of the DNAns participate in the network, and a few of them form small clusters. Therefore, the degree of connectivity of the network is large. This can also be shown by computing the number ($C_s$) of DNAns that are part of the network's largest component. When $C_s = N$, only one cluster in the system is formed by the connection of all DNAns. In contrast, nanostars are split into clusters when the value of $C_s < N$. In the inset of Fig. S8, we show the time evolution of $C_s(t)/N$. We note that at all concentrations, the system reaches a plateau in which $C_s(t)/N \simeq 1$, indicating that nanostars are part of a single cluster spanning the size of the system. This is also reported in the main plot of Fig. S8, where we show that at the steady-state $C_s/N \simeq 1$ for $\rho \geq 0.02$.

### Shortest path between branching points

In graph theory, the shortest path from a source vertex to a target vertex is the path with the minimum number of edges between them. We use Mathematica [23] to find the shortest path connecting any two branching points in a network diagram. In Fig. S9(a), we give an example of this analysis for two branching points. As a result, we obtain the molecule IDs (labels) of the DNAns in the shortest path, which allows us to identify how this path looks in our simulations (see Left panel in Fig. S9(b)). Since we use periodic boundary conditions, an object which has passed through one face of the simulation box re-enters through the opposite face. Therefore, it is necessary to use the minimum image criterion (MIC) to reconstruct the shortest path (Right panel in Fig. S9(b)). For simplicity and computational efficiency, we reconstruct the shortest path and determine its radius of gyration ($R_{g,\lambda}$) by using only the core beads of nanostars:

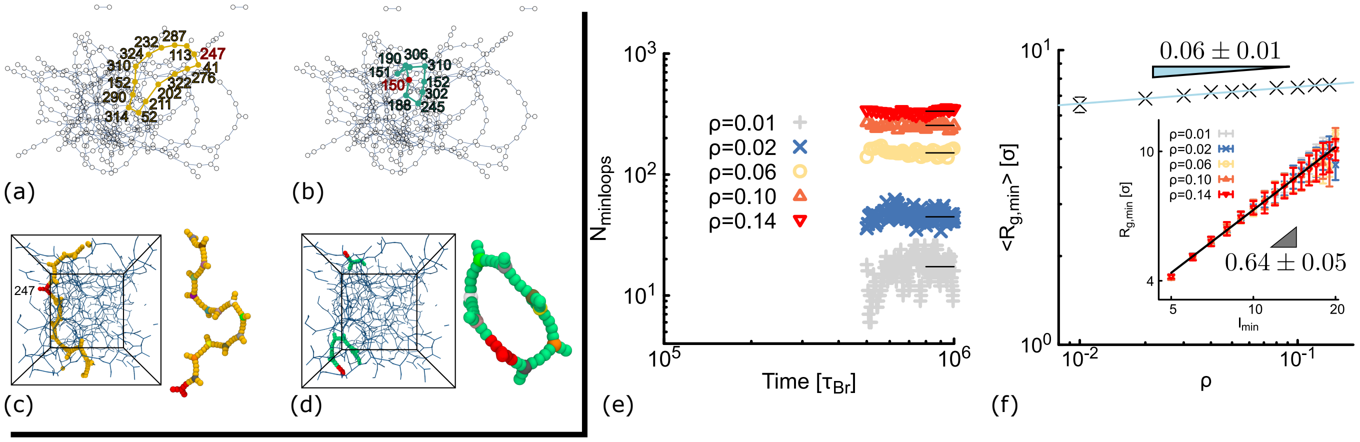


Figure S10. **Minimum loops analysis.** **(a)-(b)** Network diagram for the system with $\rho = 0.02$ and at $t = 10^6\tau_{Br}$ (same as the one in Fig. S7(a)). The minimum loop (highlighted in yellow (or green)) that passes through vertex 247 (or 150) (highlighted in red) is shown. The size of this minimum loop is $l_{min} = 15$ (or 9) nanostars. Labels depict the molecule IDs of nanostars. **(c)** Left panel shows the configuration from the simulation of the network diagram in (a). We use the minimum image criterion to reconstruct the correct path (right panel). To ease visualisation, the core beads of nanostars in the path are highlighted. **(d)** shows analogous results for the network diagram in (b). **(e)** Time evolution of the total number of minimum loops computed from simulations at times $t \geq 5 \times 10^5\tau_{Br}$ after turning on the attraction between patches. **(f)** Scaling of the average radius of gyration of the minimum loops as a function of $\rho$. Error bars represent the SEM and they are smaller than the symbol size. Inset shows the plot of the radius of gyration (computed using Eq. S10) as a function of the size ($l_{min}$) for different concentrations of nanostars. Results in this panel are obtained from the analysis of at least 25 independent gel configurations at each volume fraction.

$$R^2_{g,\lambda} = \frac{1}{\lambda}\sum_{n=1}^{\lambda}\left[\boldsymbol{r}_{\text{mean}} - \boldsymbol{r}_n\right]^2, \qquad \text{(S9)}$$

where $\boldsymbol{r}_n$, $\boldsymbol{r}_{\text{mean}}$ and $\lambda$ represent the position of the $n$-th core-bead of a DNAns in the shortest path, the centre of mass of the shortest path and its length, respectively. By repeating this process at different times after full network formation and at different concentrations, we obtain the distribution $P(\lambda)$ of the number of DNAns in the shortest path between branching points. This distribution is well fitted by a Gaussian (Fig. S9(c)). From this fit we extract the average of the distribution $\langle\lambda\rangle$ reported in Fig. S9(d). In marked contrast with typical results from chemical cross-linking gels, we discover that the average path length $\langle\lambda\rangle$ *increases* with DNAns concentration, with a similar exponent (0.1) found for the growth of minimum loop length (see Fig. **2**(f) in main text). This is also in stark contrast with the intuition that a larger density of building blocks in a network would favour the creation of more branching points and hence shorter minimum paths between any two branching nodes.

In line with these results, we also find that the average radius of gyration of the shortest paths in between any two nodes, $R_{g,\lambda}$, grows with the concentration as $\langle R_{g,\lambda}\rangle \sim \rho^{0.06}$ and therefore $\langle R_{g,\lambda}\rangle \sim \langle\lambda\rangle^{0.6}$, suggesting that the shortest paths adopt conformations compatible to those of linear chains in a good solvent (see Fig. S9(e)). Whilst this is in agreement with the increase of $\langle\lambda\rangle$ described above, it is in dramatic contrast with both polymeric systems and colloidal gels, where $R_{g,\lambda}$ decreases as the concentration of the building blocks increases [24].

We also computed the average radius of gyration of the shortest path as a function of $\lambda$. Results for different concentrations are shown in Fig. S9(f). These results are compatible with the Flory exponent for self-avoiding walks, i.e., $\nu = 0.58$, and are also in line with our previous findings from the scaling of $\langle R_{g,\lambda}\rangle$ and $\langle\lambda\rangle$. The fractal dimension of the shortest paths is given by $d_f = 1/\nu = 1.7$. It is worth stressing here that the scaling reported in Fig. S9(f) appears to be independent of the concentration of nanostars, and so is $d_f$. This suggests that the network structure does not change its fractal dimension despite displaying different material behaviours (from liquid-like at $\rho = 0.01$ to gel-like at $\rho = 0.14$, see MSDs in Fig. S5(g)). This also suggests that there may be a different motif in the network that emerges when the gel changes its physical behaviour.

## Minimum loops

In the network diagrams, a loop is a closed path with no repetition of vertices or edges other than the starting and end points. Here, we compute all the loops ($N_{i,l}$) with size $l \in [4, l_m]$ nanostars, passing through the ith-vertex in a graph (note that $i \in [1, N]$). Since the number of loops $N_{i,l}$ increases exponentially with $l_m$, we restrict the search to only those loops formed by at the most

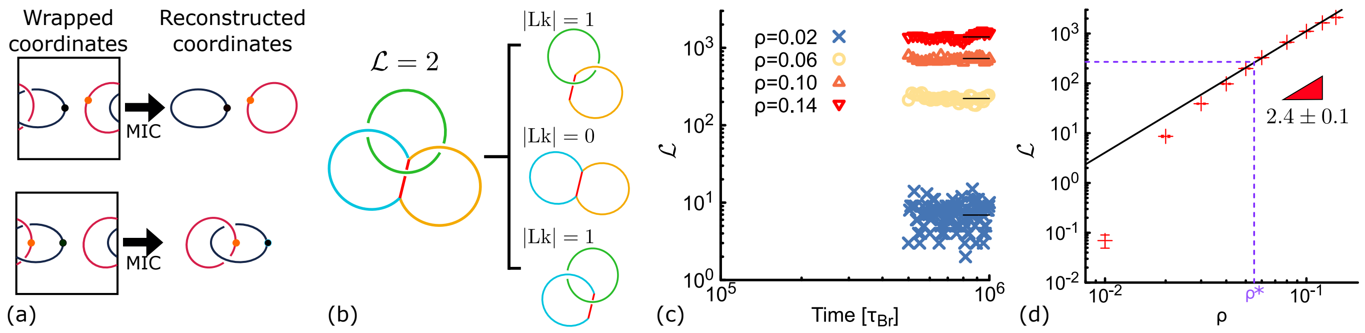


Figure S11. **Linking number analysis.** **(a)** Sketch of the reconstruction of two minimum loops using the minimum image criterion (MIC). The first particle in the set of beads in a minimum loop is represented by a dot (black and orange for the two different rings). The top panel shows the case where the reconstructed loops are not linked. In the bottom panel, a permutation that preserves the sequence of beads in the red minimum loop is applied. This is reflected in the change of the orange dot's position (with respect to the top panel) before applying the MIC. This time the reconstructed loops capture the correct linking between them. **(b)** Sketch of the linking number computed when two rings (the cyan and yellow) share vertices (region depicted in red) and interact with a third ring (in green). **(c)** Temporal evolution of the total linking number ($\mathcal{L}$), computed for different concentrations of DNAns. **(d)** Scaling of $\mathcal{L}$ with $\rho$. Results are expressed as mean ± SEM, obtained from at least 25 independent gel configurations per volume fraction. Error bars are smaller than symbols.

$l_m = 20$ nanostars. The loop with the smallest size (created by the least number of DNAns, $l_{min}$) is the minimum loop passing through vertex $i$. We note that two or more vertices in the same graph could share a minimum loop; therefore, our algorithm ensures to exclude repeated minimum loops.

In Fig. S10(a) and (b), we show examples of this analysis for two different vertices in the same graph, $i = 247$ and $i = 150$, respectively. First, we obtain the molecule IDs of all nanostars in the minimum loops passing through those vertices using Mathematica graph analysis tools (corresponding snapshots from simulations are reported in panels (c) and (d)). We observe that in the former case, the minimum loop identified in the graph is actually a linear path (closed through the periodic boundary conditions and thus an artefact of the simulation setup), and that is formed by $l_{min} = 15$ nanostars. We avoid considering these paths by using: first, the minimum image criterion to reconstruct the minimum loops, and then, we impose the condition $R_{g,min} < 0.3L = 12\sigma$ on the radius of gyration of the minimum loop:

$$R^2_{g,min} = \frac{1}{l_{min}} \sum_{n=1}^{l_{min}} [\boldsymbol{r}_{\rm mean} - \boldsymbol{r}_n]^2 \,, \qquad \text{(S10)}$$

this time $\boldsymbol{r}_n$, $\boldsymbol{r}_{\rm mean}$ and $l_{min}$ represent the position of the $n$-th core-bead of a DNAns in the minimum loop, its centre of mass and its size, respectively. Figure S10(d) shows a successfully identified minimum loop from our simulations, formed by $l_{min} = 9$ DNA nanostars.

By repeating this procedure for all the vertices in a graph (not only the branching points) and then for different time-steps, we obtain the temporal evolution of the total number of minimum loops ($N_{minloops}(t)$) that is reported in Fig. S10(e). At large times, $N_{minloops}(t)$ reaches a plateau for all concentrations, from which we compute the average and SEM of the number of minimum loops. This information is then used to produce Fig. **2**(f) (inset) in the main text, and we find the scaling $N_{minloops} \sim \rho$. We also obtain the distribution $P(l_{min})$ of the number of DNAns in the minimum loop depicted in Fig. **2**(e) of the main text, and the average of the distribution $\langle l_{min} \rangle$ (see Fig. **2**(f)). Likewise, here we obtain the average radius of gyration in Fig. S10(f). The scaling exponents, $\langle R_{g,min} \rangle \sim \rho^{0.06}$ and $\langle l_{min} \rangle \sim \rho^{0.1}$, are consistent with our previous results from the analysis of the shortest path between branching points, $\langle R_{g,min} \rangle \sim \langle l_{min} \rangle^{0.6}$.

We also computed the average radius of gyration of the minimum loop as a function of $l_{min}$. Results for different concentrations are shown in the inset of Fig. S10(f), from which we obtain $\nu = 0.64$. These results confirm that $d_f = 1/\nu$ is independent of the concentration of nanostars, just as we found for the shortest path.

### Linking number

The linking number between a pair of closed-oriented curves $\gamma_i$ and $\gamma_j$ can be computed through the numerical integration of the double integral:

$$\mathrm{Lk}(\gamma_i, \gamma_j) = \frac{1}{4\pi} \oint_{\gamma_i} \oint_{\gamma_j} \frac{(\mathbf{r}_j - \mathbf{r}_i)}{|\mathbf{r}_j - \mathbf{r}_i|^3} \cdot (\mathrm{d}\mathbf{r}_j \times \mathrm{d}\mathbf{r}_i), \qquad \text{(S11)}$$

where $\mathbf{r}_i$ and $\mathbf{r}_j$ are the vectors defining the position of all the points along the curves $\gamma_i$ and $\gamma_j$, respectively. In our simulations, these curves are constructed from the set of beads in the minimum loops (not only core beads)

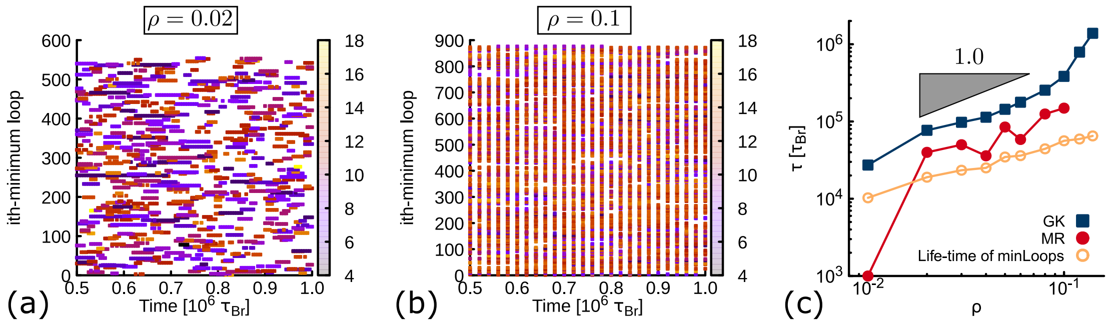


Figure S12. **Minimum loops life-time**. **(a)-(b)** Kymographs of the minimum loops appearance (colour when the loop is present in the simulation and white when it is not) at $\rho = 0.02$ and $\rho = 0.1$, respectively. Colour map represents the length of minimum loops in the system, made by $l \in [4-18]$ DNAns. **(c)** Plot comparing the scaling of the relaxation time measured from GK and MR simulations, with the average life-time of minimum loops.

identified from the analysis described in the previous section.

It is important to mention here some technical aspects that arise during the computation of the linking number in our system. (i) Since we reconstruct minimum loops (formed through the PBC) using the minimum image criterion, the linking between two rings could be overlooked. This happens when the initial beads in the two paths are not on the same side of the simulation box (see Fig. S11(a)). To account for the correct linking number, we perform the following analysis: After identifying the IDs of the set of beads that form two minimum loops and reconstructing them using the MIC, we compute their linking using Eq. S11. If Lk $= 0$, we reconstruct the second minimum loop starting from a different bead, but keeping the order of particles in the loop (the *rotate function* in C++ is a good example of how to implement this). We continue with this calculation until either Lk $\neq 0$ or we have performed all the possible rotations of the set of beads in the second minimum loop. (ii) We note that two minimum loops can share some vertices (see Fig. S11(b)); this leads to numerical errors in the calculation of the linking number. Therefore, we use only pairs of disjoint rings which do not share vertices when computing Lk. (iii) The orientation of the minimum loops is randomly assigned due to the undirected nature of our graphs. Therefore, the value of Lk averaged over all the pairs of minimum loops is close to zero. However, to rationalize the elasticity observed in our simulations and measure how "entangled" the system is, we count the total number of times that two minimum loops are linked regardless of the sign of Lk:

$$\mathcal{L} = \sum_{i>j}^{N_{minloops}} |\mathrm{Lk}(\gamma_i, \gamma_j)| \,. \qquad \text{(S12)}$$

In Fig. S11(c), the time evolution of $\mathcal{L}(t)$ is shown. We observe that $\mathcal{L}(t)$ reaches a plateau at long times from which we compute the average and SEM of the total linking between minimum loops. This information is then used to produce Fig. S11(d) where we show the value of $\mathcal{L}$ at the plateau against concentration, from which the scaling $\mathcal{L} \propto \rho^{2.4}$ for $\rho$ larger than the overlapping volume fraction, $\rho^* \sim 0.056$ (see Section VI), is found.

### Average life-time of minimum loops

Here we compute the average life-time of minimum loops to provide direct evidence of their contribution to the viscoelastic properties of limited valence networks. First, we proceed to label minimum loops from 1 to $\mathcal{N}$, with $\mathcal{N}$ the total number of unique loops during the whole simulation. In general, it is expected that $\mathcal{N} > N_{minloops}$, meaning that although the total number of minimum loops ($N_{minloops}$) remains almost constant during the simulation, different DNAns are forming the loops. In Figs. S12(a,b) we show kymographs capturing the appearance of each unique minimum loop in time. Then, we use a cluster algorithm (of nearby values in time) to find the time that minimum loops existed continuously in the simulation. The average size of the clusters represents the average life-time of minimum loops. In Fig. S12(c) we show that the relaxation time obtained from GK and MR simulations exhibits the same scaling with volume fraction as the average life-time of minimum loops. Hence we conclude that the dynamics of minimum loops drives the relaxation time of the system. At timescales shorter than the relaxation time of the fluid, the topology of the network is fixed and minimum loops are the main drivers of elasticity. At timescales larger than the relaxation time, the network topology changes and the fluid becomes liquid dominated instead of solid dominated. The viscosity of these fluids is not controlled by the topological motives.

## VIII. INTERPENETRATION FROM EXPERIMENTS

Our experiments aim to showcase the interpenetration in DNAns hydrogels by using two distinct types of 3-armed nanostar design: DNS-A and DNS-B. The core sequence of DNS-B is obtained by shuffling some base pairs of the DNS-A core so the two designs have the same composition and melting temperature but different sequences (refer to Table S3). The different core sequences are needed to avoid adhesion effects between the two types of nanostars due to the imperfect particle self-assembly and formation of unintended cross-linkers [25]. Still, we preserve their flexibility by keeping the unpaired adenosines at the core of the Y-shaped structure and preceding the overhangs. To ensure that only nanostars of the same type bind together when they are mixed, distinct sequences are designed for the sticky ends, each comprising 6 nucleotides, having equal self-binding strength: $\Delta G$ is -8.5 kcal/mol (at [NaCl]= 150mM) and -9.3 kcal/mol (at [NaCl]= 500mM) for DNS-A, while for DNS-B $\Delta G$ is -8.2 kcal/mol (at [NaCl]= 150mM) and -9.0 kcal/mol (at [NaCl]= 500mM). As expected, elevating the salt concentration makes the binding between nanostars energetically more favourable because of the reduced repulsion amongst negatively charged DNA bases. Consequently, it is shown an elevation of the melting temperature of the sticky ends with the salt concentration (see Table S4). These values are obtained using the analysis tool from NUPACK [26]. Knowing the $T_{m2}$ was essential to determine the optimal conditions for our future experiments. To identify each design when mixed together, a specific dye molecule was introduced at a 9-nucleotide distance from the sticky end of one of three oligos: i6-FAMK for DNS-A and iCy3 for DNS-B. The oligos sequences were designed via NUPACK and purchased with the dye modifications from IDT.

The DNS-A and DNS-B stock solutions (at 1mM) are prepared as described in section II but in two separate test tubes to prevent the formation of unintended secondary structures during the annealing step. In both cases, the fluorescently tagged and untagged oligomers are mixed at a molar ratio of 1:10 (or 1:20). From the nanostar stock solutions, we prepared samples at lower concentrations to investigate the contribution of interpenetration under three different conditions: inside (100$\mu$M), in proximity (250$\mu$M) and outside (400$\mu$M and 500$\mu$M) the phase separation region. Here, we report the sample preparation at final concentration [DNS-A]= 250$\mu$M and [DNS-B]= 250$\mu$M in a total volume of 10$\mu$L. We start by placing a test tube in a heat block set at 60°C and adding: (i) 5$\mu$L of Nanostar Buffer (at 150mM or 500mM NaCl, depending on the experiment). (ii) 2.5$\mu$L from DNS-A and DNS-B stocks both pre-heated at 60°C for 2 min. The solution is well mixed by pipetting 3-5 times via pre-heated tips; then it is heated at 60°C for another 2 min, and finally, 5$\mu$L of the sample is loaded on the slide.

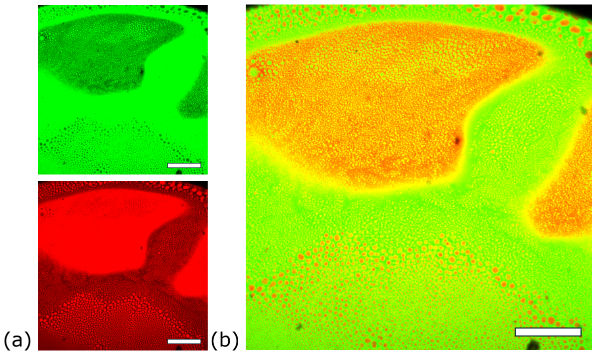

Figure S13. **Confocal image** of [DNS-A] and [DNS-B] at 500$\mu$M in 150mM NaCl. The image, taken after 240 min of sample sedimentation, shows the green and red channels split in **(a)** and then merged in **(b)**. The scale bars are 250 $\mu$m in all images.

The samples are visualised with a Zeiss LSM700 confocal microscope using a 20x (Numerical Aperture = 0.4) objective. As in microrheology experiments, the sample is left to equilibrate at 25°C for 5 minutes by placing a stage-top thermal chamber on the microscope stage.

We captured z-stack images (512x512 pixels, with a zoom=0.5) over time with an interval of 30 min for 10-17 h. We used a scan time of 15.49 s and a laser intensity equal to 2.4% (no bleaching effects were detected at this intensity value). During the image acquisition, the 488 nm and the 555 nm lasers scan the sample sequentially to excite FAMK (DNS-A, shown in green in the top left of Fig. S13(a)) and Cy3 (DNS-B, shown in red in the bottom left of Fig. S13(a)), respectively. To visualise the final image, we assemble the two channels into one via Fiji [27]. As one can observe in Fig. S13(b), when [DNS-A]= 500$\mu$M and [DNS-B]= 500$\mu$M at [NaCl]= 150mM, the image is partitioned into three dominant colours: green, red and yellow. Whilst it is evident the green

| | Segment I | FJC | Segment II | FJ | Sticky end |
|---|---|---|---|---|---|
| **DNS-A** | | | | | |
| 5′– | CTGGATCCGCGGAAGCTTAA | AA | CGGAATTCGCATGGATCCCC | A | CGATCG –3′ |
| 5′– | CTGGATCCGCGGAAGCTTAA | AA | CGGAATTCGCA/i6-FAMK/GGATCCCC | A | CGATCG –3′ |
| 5′– | GGGGATCCATGCGAATTCCG | AA | CTGAATTCCCTGGGATCCCG | A | CGATCG –3′ |
| 5′– | CGGGATCCCAGGGAATTCAG | AA | TTAAGCTTCCGCGGATCCAG | A | CGATCG –3′ |
| **DNS-B** | | | | | |
| 5′– | GTCAATGCCCGCGGGCAATT | AA | GGGCATGGAATTCCGCATCC | A | GCTAGC –3′ |
| 5′– | GTCAATGCCCGCGGGCAATT | AA | GGGCATGGAATT/iCy3/CCGCATCC | A | GCTAGC –3′ |
| 5′– | GGATGCGGAATTCCATGCCC | AA | CCCTTGGGAATTCGATCCCG | A | GCTAGC –3′ |
| 5′– | CGGGATCGAATTCCCAAGGG | AA | AATTGCCCGCGGGCATTGAC | A | GCTAGC –3′ |

Table S3. Strand sequence used in the design of DNS-A and DNS-B with valence $f = 3$ for confocal experiments. For each nanostar type, the first and second row of the table shows the same oligo sequence, which differs only in the presence of the molecule dye inserted in Segment II. The oligo with the molecule dye encoded is used in the sample preparation in a ratio of 1:10 (or 1:20) concerning the unlabelled oligo.

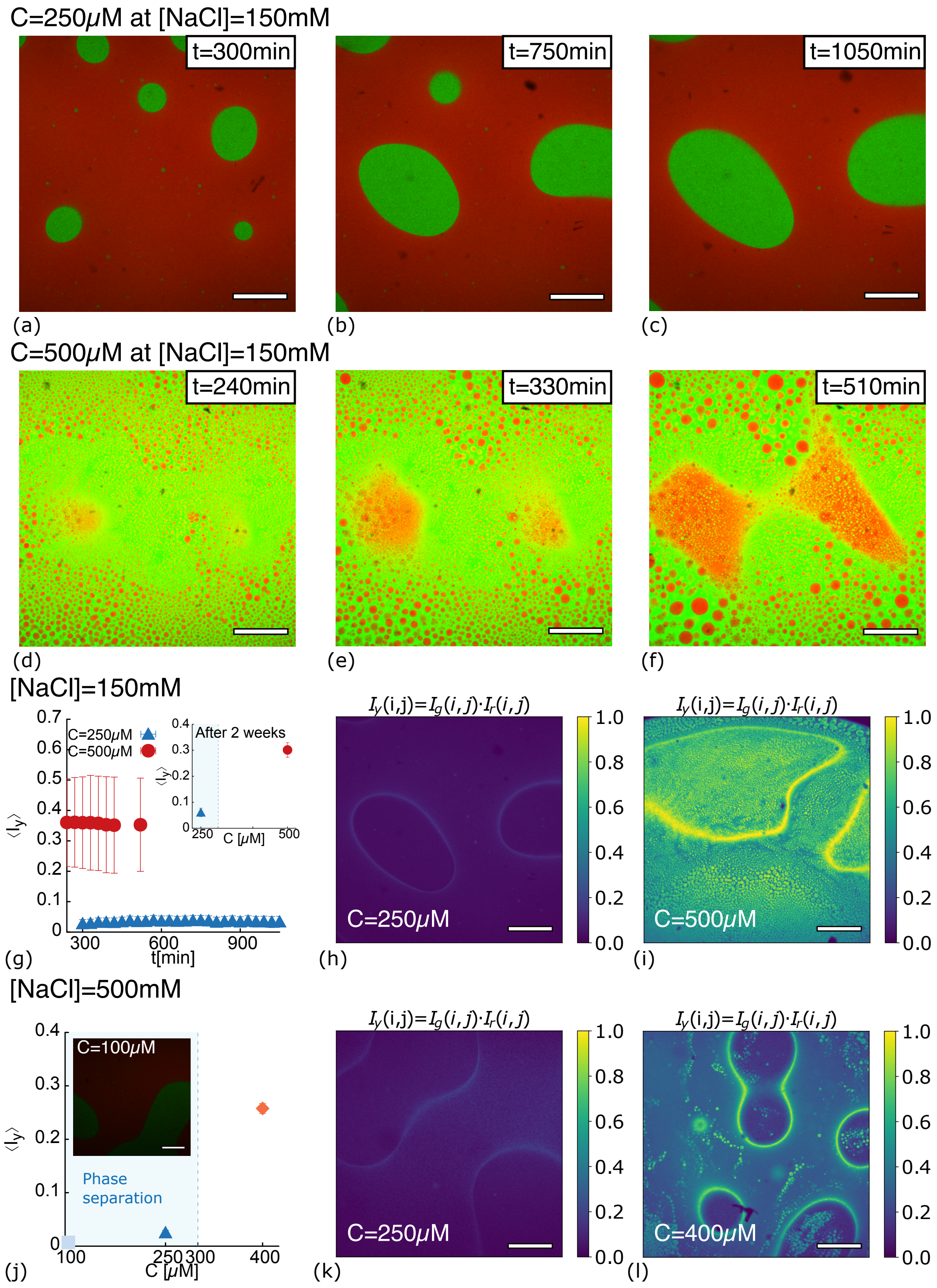


Figure S14. **Interpenetration analysis** Snapshots taken at different time points display the system's progression at DNA concentrations of 250$\mu$M **(a)-(c)** and 500$\mu$M **(d)-(f)** and at 150mM NaCl. **(g)** Average intensity of the yellow component ($\langle I_y \rangle$) plotted as a function of time (in minutes) for C= 250$\mu$M (blue triangle dots) and C= 500$\mu$M (red circle dots) and at 150mM NaCl. Each data point represents the mean $\pm$ SD of the yellow intensity computed across the image for a fixed time-point. The inset shows the mean $\langle I_y \rangle \pm$ SD of the samples from at least 3 images taken after two weeks. **(h)-(i)** panels show the product matrix at 150mM NaCl for C= 250$\mu$M and C= 500$\mu$M, respectively. **(j)** Shows the value of $\langle I_y \rangle$ for samples at 500mM NaCl and at C=100, 250 and 400$\mu$M. Each data point represents the mean $\pm$ SD of the yellow intensity calculated by averaging the mean intensity values over 3 different time points. Inset shows a confocal image of a sample with C= 100$\mu$M. **(k)-(l)** panels show plots of the product matrix at 500mM NaCl for C= 250$\mu$M and C= 400$\mu$M, respectively. Scale bars in all images are 250 $\mu$m. For all panels in this figure, each independent sample mixture was prepared once, from the same pure batches of DNS-A and of DNS-B, following the protocol in Supplementary Section VIII.

| Sequence | [NaCl]=150mM | | [NaCl]=500mM | |
|---|---|---|---|---|
| | 100$\mu$M | 500$\mu$M | 100$\mu$M | 500$\mu$M |
| **A**:5′−CGATCG−3′ | 31°C | 38°C | 37°C | 44°C |
| **B**:5′−GCTAGC−3′ | 30°C | 37°C | 36°C | 43°C |

Table S4. Melting temperatures of the sticky ends for DNS-**A** and DNS-**B** at [NaCl] equal to 150mM and 500mM by varying the DNA concentration from 100$\mu$M to 500$\mu$M; inside and outside the phase separation region respectively.

component refers to the presence of DNS-A and the red one to DNS-B, the yellow part indicates the presence of regions/pixels populated by both types of nanostar. We interpret the abundance of yellow regions as signs of interpenetration. The yellow regions are seen mainly at the edges of red/green droplets where the two phases are in touch.

In Fig. S14, snapshots show the temporal evolution from confocal images of systems at [NaCl]= 150mM. When C= 250$\mu$M, panels (a)-(c) reveal there is no overlap between the green and red signals, indicating a complete demixing of the two components over time. On the other hand, the snapshots for C= 500$\mu$M in panels (d)-(f) show that the mixed "yellow" regions persist and do not undergo demixing throughout the observation period. To better quantify the degree of interpenetration at different concentrations, we first split the images into two distinct channels and extract a 512x512 intensity matrix for the green ($I_g$) and one matrix for the red ($I_r$) channel. The elements of each matrix are integer numbers referring to the pixel values, which span from 0 to 255 (maximum value for an 8-bit image). Afterwards, we subtract the noise, obtained by taking an image with the same microscope settings (laser intensity, gain, pinhole size, etc.) on an empty slide, and set any negative value to zero. We then normalise the elements of both matrices by 255. Finally, to quantify the degree of mixing, we perform an element-wise multiplication consisting of multiplying each element of $I_g$ with the corresponding element in $I_r$, as $I_g(ij) \cdot I_r(ij)$, to obtain a resulting matrix of dimension 512x512 ($I_y$). A first qualitative analysis was conducted by plotting a two-dimensional map of the $I_g$, $I_r$, and $I_y$ matrices. For example, in Fig. S14(h-i), we plot the $I_y$ matrix for different sample conditions: low values of $I_y(ij) \simeq 0$ (in dark violet) represent the presence of one of the 2 components, while high values of $I_y(ij) > 0$ (green to yellow) represent regions where the contribution of both channels is high. We highlight that values of the yellow product matrix are overall high at high concentrations of DNAns (500$\mu$M, see Fig. S14(i)) and overall very small in the case of low concentration of DNAns (250$\mu$M, see Fig. S14(h)). Increasing the salt concentration to [NaCl]= 500mM produces similar results (Fig. S14(k-l)).

From the $I_y$ matrix, we compute the average and the standard deviation across all entries to measure the overall extent of the yellow (interpenetration) signal across the image. In Fig. S14(g), the quantity of interest is denoted as $\langle I_y \rangle$, and it was computed for each image taken at a 30-minute interval. As one observes from the plot, the intensity of the yellow regions remains constant over time, displaying an average interpenetration of $\langle I_y \rangle \simeq 0$ for C= 250$\mu$M and of $\langle I_y \rangle \simeq 0.4$ for C= 500$\mu$M at [NaCl]= 150mM. This stability of the "yellow" regions continues over weeks, as demonstrated in the inset of Fig. S14(g). The average intensity $\langle I_y \rangle$ at [NaCl]= 500mM is reported in Fig. S14(j) for C= 100, 250 and 400$\mu$M. We confirm that the green and red components do not overlap in the phase separation region, showing $\langle I_y \rangle \simeq 0$ for C= 100$\mu$M and C= 250$\mu$M. In contrast, beyond the gel binodal at $C \leq 400\mu$M. Remarkably, we keep a greater interpenetration between the two components (Fig. S14(l))displaying an average of $\langle I_y \rangle \simeq 0.3$.

### Intensity of the self-entangled and the two-species-entangled regions

We performed this analysis using Fig. S14(f) (at [DNAns]= 500$\mu$M and t=510 minutes), in which microemulsions (red droplets inside a green area) are formed. We computed a 2D-map of "yellow signal" (see Fig. S15(a)) where low (high) values of $I_y(i,j)$ represent regions with unbalanced (balanced) concentrations of the two species.

We proceed to identify three droplets with different areas covered by yellow color. For these droplets, we computed the average of the green and red intensities at a fixed distance ($i$) in the 2d-map: $\langle I_g(i) \rangle = \frac{1}{n}\sum_{j=1}^{n} I_g(i,j)$ and $\langle I_r(i) \rangle = \frac{1}{n}\sum_{j=1}^{n} I_r(i,j)$. Results are shown in Figs. S15 (b)-(d), we note that the average red intensity in the middle ($\langle I_{r,max} \rangle \sim 230$) and outside the droplets is different ($\langle I_{r,min} \rangle \sim 90$ for droplet 1, $\sim 80$ for droplet 2, and $\sim 110$ for droplet 3). Therefore, the red intensity ratio between the maximum (inside) and the minimum (outside) intensities observed is: 2.5, 2.8 and 2.1 for the three droplets, respectively. An analogous behaviour is observed for the green intensity, however, since the image from experiments is 8-bits, the maximum intensity we can resolve is 255. These results suggest that density of DNAns, either red or green, is different inside and outside the droplets. Yet, the sum of both signals must be constant across the image.

### Temperature variations

To monitor the mixing/demixing behaviour of the binary DNAns system, we performed an in situ annealing/quenching on a sample by varying the temperature in the incubator chamber while recording images of the

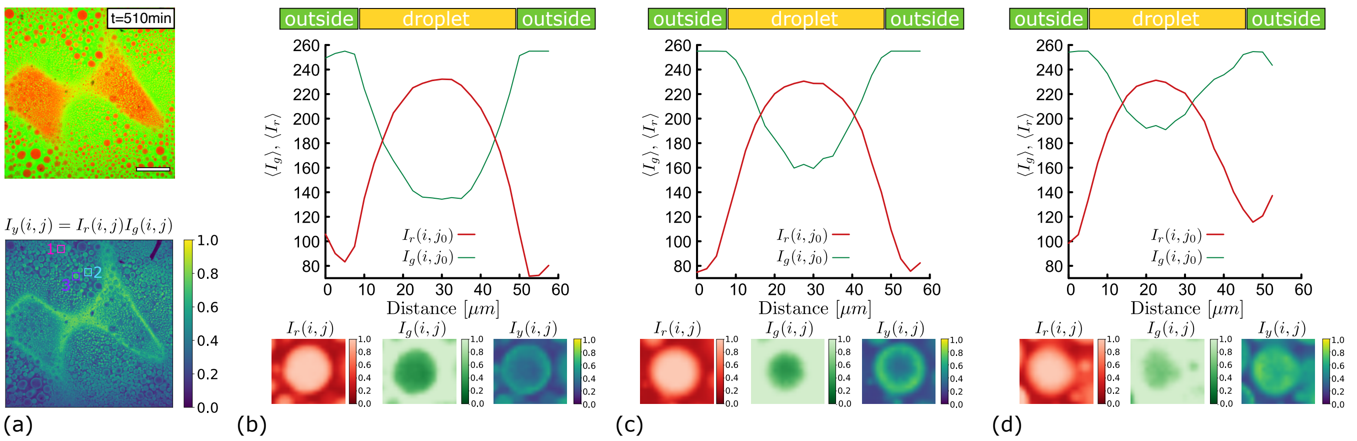


Figure S15. **Intensity comparison outside and inside droplets. (a)** Top panel shows snapshot obtained from confocal experiments after 510 minutes of mixing [DNS-A]= 500$\mu$M and [DNS-B]= 500$\mu$M. Bottom panel shows pixel-by-pixel product (or yellow signal). **(b)-(d)** Results for three different droplets selected from panel (a). The images at the bottom show droplets when looking at the red channel (Cy3, 555 nm), green channel (FAMK, 488 nm) and the yellow map. Main plot shows the intensity profile of the red and green signals. These are obtained from the $I_r(i,j)$ and $I_g(i,j)$ maps by averaging the red and green intensities at a fixed horizontal distance in the image.

sample. Our first attempt involved DNS-A and DNS-B at a concentration of 500$\mu$M with 150mM NaCl. To start, we heated the sample to 60°C, which is above the melting temperature ($T_{m2}$) of the stick ends. This allows the molecules to diffuse freely in the solution. We then let the sample equilibrate for 15 minutes. Next, we adjusted the thermal chamber to 25°C to allow the nanostars to hybridise and form the network structure. The chamber temperature was tracked as the sample was cooling for over 50 minutes. We captured an image every minute throughout the heating and cooling phases until the desired temperature was achieved. As we previously explained, we extracted the $I_g$, $I_r$, and $I_y$ matrices from each picture and calculated the average ($\langle I_y \rangle$) and standard deviation of the product matrix. By plotting $\langle I_y \rangle$ against temperature (as shown in Fig. S16), we can effectively illustrate the effect of annealing/quenching on interpenetration. Our findings reveal that this in situ annealing/quenching protocol increases the extent of the interpenetration, eventually reaching 0.49 at 25°C. This final state is long-lived and displays an average interpenetration $\langle I_y \rangle \simeq 0.5$ for hours after the quenching protocol.

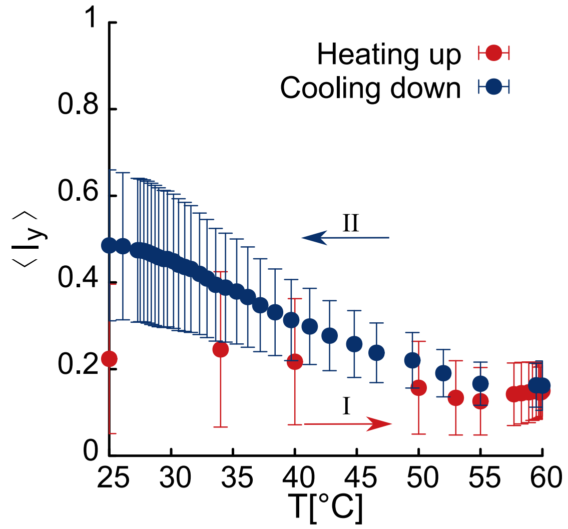


Figure S16. **Confocal results during and after temperature ramp.** This figure reports the behaviour of $\langle I_y \rangle$ in response to a temperature ramp performed with a temperature-controlled stage-top OKO Lab chamber. A single sample at C= 500$\mu$M and at 150mM NaCl was prepared (following the protocol in Section VIII) and placed on the microscope at 25°C. Then, we proceed to heat the sample to 60°C and cool it back down to 25°C. We take images during the annealing and quenching and compute the mean of the yellow intensity, $\langle I_y \rangle$, and the standard deviation across the image for each temperature explored. One can appreciate that after the in situ annealing, the cooled sample appears to display a much stronger interpenetration.

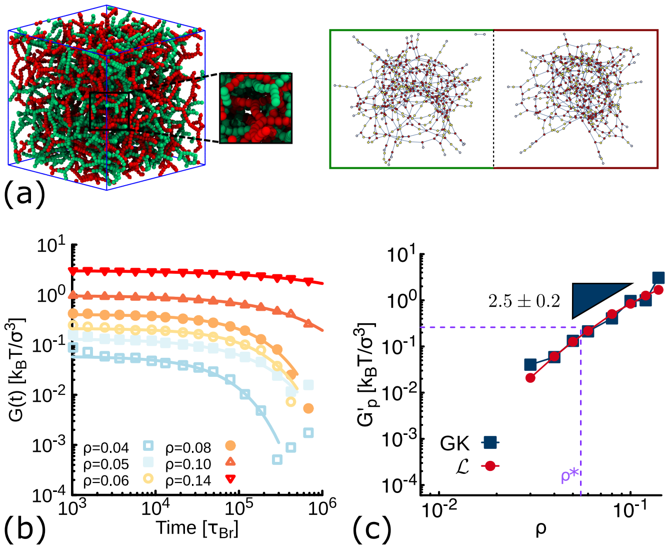


Figure S17. **Results from simulations of a binary mixture of DNA nanostars.** **(a)** Left panel shows a snapshot from simulations of a system containing two types of DNAns, A (green) and B (red) at a volume fraction $\rho = 0.05$. The attraction between patches is set such as hybridization is only allowed between A-A and B-B, but not A-B nanostars. Right panel shows the network diagram from the snapshot. The green and red squares correspond to the graph of the networks formed by only-A and only-B nanostars, respectively. **(b)** Autocorrelation of the stress-tensor obtained from Green Kubo simulations at different volume fractions. Dots represent results from simulations and lines are obtained by fitting a stretched exponential to obtain $G'_p$. **(c)** Scaling of elasticity with concentration of nanostars comparing Green-kubo simulations (dark-blue) and total linking number (red circles). Here, $\mathcal{L}$ is multiplied by a factor $8 \times 10^{-4}$ to help comparison.

## IX. RESULTS FROM SIMULATIONS WITH A BINARY SYSTEM OF DNANS

Here we simulate a system comprised of two types of DNAns (A and B), resembling the confocal experiments discussed in the previous section. We verified that interpenetrated structures (similar to the ones observed with only one type of DNAns), take place in the system. In Fig. S17(a) we show a typical snapshot from simulations of a binary system at a volume fraction $\rho = 0.05$, where it is clear that nanostars self-assemble into two networks (made of only-A (green) or only-B (red) DNAns), and the two networks are interpenetrated. By performing Green-Kubo simulations (Fig. S17(b)), we measure the elasticity from simulations at different concentrations of DNAns. Our analysis suggests that the elasticity of the system with two types of DNAns is of the same order of magnitude (but smaller) than the elasticity of the system with only one type of DNAns. Yet, we found the same scaling exponent for both systems, $G'_p \propto c^{2.5\pm0.2} \propto \mathcal{L}$ (see Fig. S17(c)).

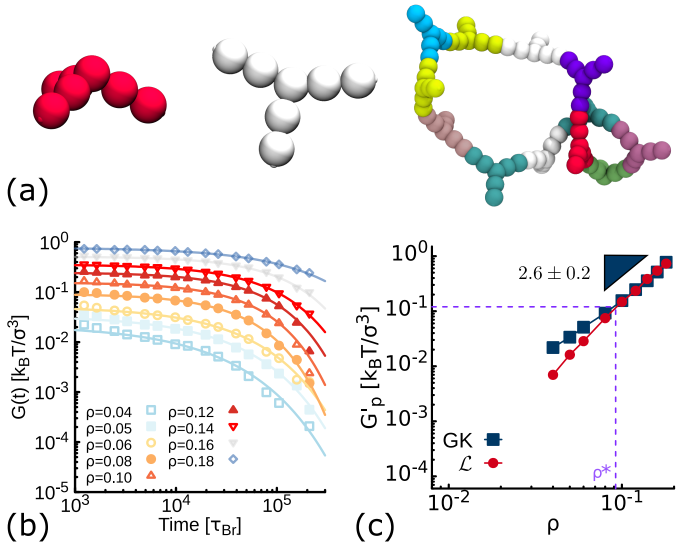


Figure S18. **Results with the non-rigid model.** **(a)** Snapshots from simulations of typical configurations of DNAns. From left to right we show a non-planar DNAns, a T-like shape DNAns and a loop. **(b)** Stress autocorrelation function obtained from Green Kubo simulations at different volume fractions. Dots represent results from simulations and lines are obtained by fitting a stretched exponential function to obtain $G'_p$. **(c)** Scaling of elasticity with concentration of nanostars comparing Green-kubo simulations (dark-blue) and total linking number betwen minimum loops (red circles). We have multiplied $\mathcal{L}$ by $2.3 \times 10^{-4}$ to help comparison.

## X. RESULTS WITH A NON-RIGID MODEL OF DNANS

In this section we check that providing DNAns with internal flexibility does not change the general results obtained with the rigid model used so far. This non-rigid model was introduced in reference [17] and it was developed to allow structural fluctuations around the Y-shaped nanostars. Briefly, harmonic and improper potentials control the angle between the dsDNA arms of a single nanostar and the planarity of the molecule, respectively (see Fig. S18(a)). The conformations explored in this model are qualitatively compatible with the conformations found in simulations with the oxDNA model for the design of DNAns used here (see Table S1). We note that since the radius of gyration of DNAns in the non-rigid model ($R = 2.11\sigma$) is smaller than the one for their rigid counterparts ($R = 2.5\sigma$), then $\rho^* = 0.09$ is larger for the non-rigid case (see Eq. S7).

We had already shown in reference [17] that allowing internal flexibility of the nanostars causes: (i) a decrease in the number of connections, (ii) an increase in the mobility of nanostars, and (iii) a decrease in the viscosity of the system. However, when looking at the scaling of elasticity with concentration of nanostars, we observe here that the results presented in the main manuscript are ro-

bust. The scaling exponent is still 2.5 (within errors), and remarkably, we confirm that above overlapping concentration the elasticity is determined by the total linking number between minimum loops (see Fig. S18(b),(c)).


[1] Um, S. H. *et al.* Enzyme-catalysed assembly of DNA hydrogel. *Nature materials* **5**, 797–801 (2006).

[2] Xing, Z. *et al.* Microrheology of dna hydrogels. *Proceedings of the National Academy of Sciences* **115**, 8137–8142 (2018).

[3] Yuan, T. *et al.* Highly permeable dna supramolecular hydrogel promotes neurogenesis and functional recovery after completely transected spinal cord injury. *Advanced Materials* **33**, 2102428 (2021).

[4] Athanasiadou, D. *et al.* Dna hydrogels for bone regeneration. *Proceedings of the National Academy of Sciences* **120**, e2220565120 (2023).

[5] Van Nguyen, K. & Minteer, S. D. Investigating dna hydrogels as a new biomaterial for enzyme immobilization in biobatteries. *Chem. Commun.* **51**, 13071–13073 (2015).

[6] Li, F., Tang, J., Geng, J., Luo, D. & Yang, D. Polymeric dna hydrogel: Design, synthesis and applications. *Progress in Polymer Science* **98**, 101163 (2019).

[7] Malouf, L. *et al.* Sculpting dna-based synthetic cells through phase separation and phase-targeted activity. *Chem* **9**, 3347–3364 (2023).

[8] Sato, Y., Sakamoto, T. & Takinoue, M. Sequence-based engineering of dynamic functions of micrometer-sized dna droplets. *Science Advances* **6**, eaba3471 (2020).

[9] Zadeh, J. N. *et al.* Nupack: Analysis and design of nucleic acid systems. *Journal of Computational Chemistry* **32**, 170–173 (2011).

[10] Biffi, S. *et al.* Equilibrium gels of low-valence dna nanostars: a colloidal model for strong glass formers. *Soft Matter* **11**, 3132–3138 (2015).

[11] Markham, N. R. & Zuker, M. DINAMelt web server for nucleic acid melting prediction. *Nucleic Acids Research* **33**, W577–W581 (2005).

[12] Conrad, N., Chang, G., Fygenson, D. K. & Saleh, O. A. Emulsion imaging of a dna nanostar condensate phase diagram reveals valence and electrostatic effects. *The Journal of Chemical Physics* **157**, 234203 (2022).

[13] Conrad, N., Kennedy, T., Fygenson, D. K. & Saleh, O. A. Increasing valence pushes dna nanostar networks to the isostatic point. *Proceedings of the National Academy of Sciences* **116**, 7238–7243 (2019).

[14] Mason, T. G. & Weitz, D. A. Optical measurements of frequency-dependent linear viscoelastic moduli of complex fluids. *Physical Review Letters* **74**, 1250–1253 (1995).

[15] Mason, T. G. Estimating the viscoelastic moduli of complex fluids using the generalized stokes-einstein equation. *Rheologica Acta* **39**, 371–378 (2000).

[16] Schmidt, R. F., Kiefer, H., Dalgliesh, R., Gradzielski, M. & Netz, R. R. Nanoscopic interfacial hydrogel viscoelasticity revealed from comparison of macroscopic and microscopic rheology. *Nano Letters* **24**, 4758–4765 (2024).

[17] Gutiérrez Fosado, Y. A. Nanostars planarity modulates the rheology of dna hydrogels. *Soft Matter* – (2023).

[18] Plimpton, S. Fast parallel algorithms for short-range molecular dynamics. *J. Comp. Phys.* **117**, 1–19 (1995).

[19] Zwanzig, R. Time-correlation functions and transport coefficients in statistical mechanics. *Annual Review of Physical Chemistry* **16**, 67–102 (1965).

[20] Ramírez, J., Sukumaran, S. K., Vorselaars, B. & Likhtman, A. E. Efficient on the fly calculation of time correlation functions in computer simulations. *The Journal of Chemical Physics* **133**, 154103 (2010).

[21] Flenner, E. & Szamel, G. Long-range spatial correlations of particle displacements and the emergence of elasticity. *Physical review letters* **114**, 025501–025501 (2015).

[22] de Gennes, P. *Scaling concepts in polymer physics* (Cornell Univ. Pr., 1979).

[23] Inc., W. R. Mathematica, Version 13.3.

[24] Rubinstein, M. *Polymer physics / Michael Rubinstein and Ralph H. Colby.* (Oxford University Press, Oxford, 2003).

[25] Jeon, B.-j., Nguyen, D. T. & Saleh, O. A. Sequence-controlled adhesion and microemulsification in a two-phase system of dna liquid droplets. *The Journal of Physical Chemistry B* **124**, 8888–8895 (2020).

[26] Hicks, L. & Santalucia, J. The thermodynamics of dna structural motifs.[j]. *Annual Review of Biophysics & Biomolecular Structure* **33**, 415–440 (2004).

[27] Schindelin et al., J. Fiji: an open-source platform for biological-image analysis. *Nature Methods* **9**, 676–682 (2012).
